# Effect of Waterlogging on Carbohydrate Metabolism and the Quality of Fiber in Cotton (*Gossypium hirsutum* L.)

**DOI:** 10.3389/fpls.2016.00877

**Published:** 2016-06-22

**Authors:** Jie Kuai, Yinglong Chen, Youhua Wang, Yali Meng, Binglin Chen, Wenqing Zhao, Zhiguo Zhou

**Affiliations:** ^1^Key Laboratory of Crop Physiology and Ecology, Ministry of Agriculture, Nanjing Agricultural UniversityNanjing, China; ^2^College of Plant Science and Technology, Huazhong Agricultural UniversityWuhan, China

**Keywords:** cotton, fiber quality, fruiting branches (FB), waterlogging (WL), carbohydrate metabolism

## Abstract

Transient waterlogging occurs frequently in the Yangtze River and adversely affects cotton fiber quality. However, the carbohydrate metabolic mechanism that affects fiber quality after waterlogging remains undescribed. Here, the effects of five waterlogging levels (0, 3, 6, 9, and 12 days) were assessed during flowering and boll formation to characterize the carbohydrates, enzymes and genes that affect the fiber quality of cotton after waterlogging. The cellulose and sucrose contents of cotton fibers were significantly decreased after waterlogging for 6 (WL_6_), 9 (WL_9_), and 12 d (WL_12_), although these properties were unaffected after 3 (WL_3_) and 6 days at the fruiting branch 14–15 (FB_14–15_). Sucrose phosphate synthase (SPS) was the most sensitive to waterlogging among the enzymes tested. SPS activity was decreased by waterlogging at FB_6–7_, whereas it was significantly enhanced under WL_3–6_ at FB_10–15_. Waterlogging down-regulated the expression of fiber *invertase* at 10 days post anthesis (DPA), whereas that of *expansin*, β-*1,4-glucanase* and endoxyloglucan transferase (*XET*) was up-regulated with increasing waterlogging time. Increased mRNA levels and activities of fiber SuSy at each fruiting branch indicated that SuSy was the main enzyme responsible for sucrose degradation because it was markedly induced by waterlogging and was active even when waterlogging was discontinued. We therefore concluded that the reduction in fiber sucrose and down-regulation of *invertase* at 10 DPA led to a markedly shorter fiber length under conditions WL_6–12_. Significantly decreased fiber strength at FB_6–11_ for WL_6–12_ was the result of the inhibition of cellulose synthesis and the up-regulation of *expansin*, β-*1,4-glucanase* and *XET*, whereas fiber strength increased under WL_3–6_ at FB_14–15_ due to the increased cellulose content of the fibers. Most of the indictors tested revealed that WL_6_ resulted in the best compensatory performance, whereas exposure to waterlogged conditions for more than 6 days led to an irreversible limitation in fiber development.

## Introduction

Cotton (*Gossypium hirsutum* L.) is known to be poorly adapted to waterlogging. Waterlogging adversely affects cotton yield and fiber quality (Hearn, 1995; Bange et al., 2004) and is therefore considered to be a major problem in global cotton production (Gillham et al., 1995). Upon maturity at 60 DAA, more than 94% of the fiber dry weight is composed of pure cellulose, a β-1,4-polymer of glucose (Basra and Malik, 1984). Many of the textile properties of cotton fibers (e.g., fiber wall thickness or maturity, strength, dye-ability, and extensibility) are directly dependent on the cellulose content (Triplett, 1993). Furthermore, the amount of another structural carbohydrate, β-1,3-glucan, rapidly increases with the onset of secondary cell wall synthesis. This carbohydrate can be broken down in the later period of fiber development to provide UDPG for cellulose synthesis (Tucker et al., 2001). Under stress conditions, more UDPG is available to participate in β-1,3-glucan synthesis (Maltby et al., 1979), and β-1,3-glucanase activity increases to counteract stress (Su et al., 2013). Sucrose, the major product of photosynthesis, can either be utilized directly via glycolysis or translocated within the plant as a soluble carbohydrate through the phloem. Following import into sink tissues, sucrose is used for the maintenance of cellular metabolism, cell wall biosynthesis, and respiration or is converted to starch for storage and used at a later time (Wind et al., 2010).

Cotton fiber formation is primarily a process of cellulose synthesis and requires many organic molecules and enzymes. Sucrose synthase (SuSy, E.C. 2.4.1.13) can both degrade and synthesize sucrose, but its primary function in cotton fiber is the degradation of sucrose to provide UDP-glucose, which is the substrate for cellulose synthesis (Delmer and Haigler, 2002; Ruan et al., 2003). In addition to SuSy, acidic/alkaline invertases (E.C. 3.2.1.26) can catalyze the hydrolysis of sucrose, providing carbon and energy for cellulose synthesis (Ruan et al., 2010). Sucrose phosphate synthase (SPS, E.C. 2.4.1.14) regulates sucrose synthesis and is considered to be the key enzyme affecting cellulose synthesis (Haigler et al., 2001).

Extensive investigations have been carried out on the enzymology of sucrose metabolism in other crops under hypoxic or anaerobic conditions caused by waterlogging. Hypoxia increases SuSy activity and expression in rice seedlings (Ricard et al., 1991). The activities of invertase, SPS, and SuSy were all higher during anaerobic growth of pondweed (*Potamogeton distinctus* A. Benn.) turions, and SuSy activity exhibited the most significant increase among these enzymes (Harada and Ishizawa, 2003). However, the activities of invertase, SPS, and SuSy were decreased in tomato fruit (Horchani et al., 2011). In addition, both cellulose and sucrose synthesis were examined in wheat roots under hypoxic conditions. Cellulose synthesis was found to be accelerated, accompanied by an increase in SuSy activity that in turn led to a change in the cell wall structure that was favorable for survival under hypoxia stress (Albrecht and Mustroph, 2003a). Our previous experiments confirmed the importance of the leaf growth during the recovery period to determine the yield of plants subjected to waterlogging (Kuai et al., 2014). However, knowledge of how the physiology of recovery affects cotton fiber quality after varying durations of waterlogging is scarce.

Based on the previous researches, we assume that the quality of fiber in cotton will be affected by carbohydrate metabolism. Specifically, gene expression related to carbohydrate metabolism changed after waterlogging which resulted in differences in enzymatic activities and substrates for cellulose synthesis. All these changes could eventually affect fiber quality. Therefore, in this study, we assess the change in fiber quality following different lengths of exposure to waterlogging and the physiological basis (i.e., sucrose metabolism) of recovery from this stress. The aim of the present study is to obtain insight into sucrose metabolism and cellulose synthesis, as well as their relationship to the fiber quality of bolls present on different fruiting branches after a period of waterlogging during flowering and boll formation.

## Materials and methods

### Plant materials and stress application

Experiments were conducted during the summer of 2011 and 2012 in ponds fitted with transparent waterproof tops at the experimental station of Nanjing Agricultural University located at Nanjing (N32°02′ and E118°50′), China. The ponds containing yellow-brown soil (Dystrudept) which was collected from (0 to 30) cm topsoil layer from the experimental station. The nutrient status of soil (0–20 cm) at that time, in terms of alkali-hydrolysable nitrogen (N), phosphorus (P), and potassium (K) concentrations, was as follows. In 2011, 63.5 mg kg^−1^ N, 20 mg kg^−1^ P, and 238.0 mg kg^−1^ K; in 2012, 67.6 mg kg^−1^ N, 14.4 mg kg^−1^ P, and 168.0 mg kg^−1^ K. Cotton seeds (cv. Siza 3) were planted on April 8 in 2011 and 2012. Individual healthy, uniform seedlings with three true leaves were transplanted into ponds (4 m in length, 4 m in width and 1.5 m in height). Each pond was planted with 5 rows, with 75 × 25 cm between plants.

The experiment comprised five waterlogging treatments (i.e., 0, 3, 6, 9, and 12 days of waterlogging) and replicated thrice in a randomized complete block design. Five soil water treatments were established on July 15—66 days after transplanting the seedlings into the ponds before the plants had been cut. Thus, the plants were actively growing and producing new flowers and boll development was occurring on lower nodes. The groups consisted of a well-watered control (WL_0_) with a relative water content maintained at 70–80% of the field capacity as well as four soil waterlogging treatments composed of waterlogging for 3, 6, 9, and 12 days (i.e., WL_3_, WL_6_, WL_9_, and WL_12_, respectively). Waterlogging was achieved by maintaining a 1-2 cm water layer on the soil surface until the evening of the 3rd, 6th, 9th, and 12th day when water was removed by opening holes at the bottom of the ponds.

White flowers on the sympodial fruiting branch at main-stem nodes 6–7, 10–11, and 14–15 of the cotton plants were tagged and defined as FB_6–7_, FB_10–11_ and FB_14–15_, respectively. Bolls were mapped by node and fruiting position. The first sympodial fruiting position closest to the main stem was designated fruiting position-1 boll. Sampling of 6 to 10 tagged position-1 bolls occurred at 17, 24, 31, 38, and 45 days post anthesis (DPA) in 2011; at 17, 24, 31, and 38 DPA in 2012; and on the day the bolls opened (BO). As suggested by Kuai et al. (2014), the initial waterlogging date and the days after the termination of waterlogging for samples on different fruiting branches, which reflected the developmental stage of the boll relative to the timing of induced hypoxia, are shown in Table 1.

**Table 1:** **Initial waterlogging date and days after terminating waterlogging for samples on different fruiting branches in 2011 and 2012**.

**Fruiting**	**2011**						**2012**					
**branches**	**Initial waterlogging date for samples**	**Days post anthesis (d)**	**Days after terminating waterlogging (d)**				**Initial waterlogging date for samples**	**Days post anthesis (d)**	**Days after terminating waterlogging (d)**			
			**WL_3_^a^**	**WL_6_**	**WL_9_**	**WL_12_**			**WL_3_**	**WL_6_**	**WL_9_**	**WL_12_**
FB_6–7_	–7 DPA^b^	17	21	18	15	12	–2 DPA	17	16	13	10	7
		24	28	25	22	19		24	23	20	17	14
		31	35	32	29	26		31	30	27	24	21
		38	42	39	36	33		38	37	34	31	28
		45	49	46	43	40		ND^c^				
FB_10–11_	–16 DPA	17	30	27	24	21	–12 DPA	17	26	23	20	17
		24	37	34	31	28		24	33	30	27	24
		31	44	41	38	35		31	40	37	34	31
		38	51	48	45	42		38	47	44	41	38
		45	58	55	52	49		ND				
FB_14–15_	–36 DPA	17	50	47	44	41	–26 DPA	17	40	37	34	31
		24	57	54	51	48		24	47	44	41	38
		31	64	61	58	55		31	54	51	48	45
		38	71	68	65	62		38	61	58	55	52
		45	78	75	72	69		ND				

a *WL_3_, WL_6_, WL_9_ and WL_12_ stand for waterlogging for 3, 6, 9, and 12 days, respectively*.

b *“+” (after) and “−” (before) DPA represent initial waterlogging date for the samples*.

c *ND, no data as bolls opened before 45 DPA in 2012*.

For each time point, the boll wall, seeds, and fiber of the sampled bolls were separated and the fibers were immediately placed in liquid nitrogen and stored at −80°C until the measurement of enzyme activity and gene expression.

### Measurements of fiber quality

Tagged bolls were hand-harvested after opening and were ginned in individual groups according to fruiting branch. Ginned fiber from each group was sent to the Cotton Quality Supervision, Inspection and Testing Center of the China Ministry of Agriculture for quality analysis. Fiber quality, including the fiber upper-half mean length (UHML) and strength of each lint sample, was assessed by a High Volume Instrument at the Cotton Quality Supervision, Inspection and Testing Center of the China Ministry of Agriculture.

### Sucrose, cellulose and β-1,3-glucan assessment

Fibers were digested with an acetic-nitric reagent, and the cellulose content was measured with anthrone according to the method described by Updegraff (1969).

Sucrose was extracted and quantified by the Pettigrew method with modifications (Pettigrew, 2001). Approximately 0.3-g dry weight (DW) fiber samples were extracted via three successive 5-ml washes of 80% ethanol. The ethanol samples were incubated in an 80°C water bath for 30 min. The samples were then centrifuged at 10,000 × g for 10 min, and three aliquots of supernatant were pooled together for the sucrose measurement.

β-1,3-glucan was measured according to Albrecht's method (Albrecht and Mustroph, 2003b). Fiber tissues (0.3-g dry weight) were extracted in 5 ml of 80% ethanol containing 10 mM EDTA for 2–3 h to remove fluorescent compounds. The samples were homogenized in 6 ml of 1N NaOH, incubated for 30 min at 80°C, and centrifuged for 15 min at 380 rpm. Six-hundred microliters of supernatant was adjusted to 1.2 ml and neutral pH with a 0.1% anilineblue solution and 0.63 ml of 1 N HCl. Then, 1.77 ml of 1 M glycine/NaOH buffer (pH 9.5) was added. The mixture was incubated for 20 min at 50°C and returned to room temperature for 30 min. The fluorescence of the callose-aniline blue complex was measured with a fluorimeter using 400 nm excitation light and 510 nm emission light.

### Fiber enzymatic extraction and analysis

The enzyme extraction and assay of SuSy, invertases and SPS activity was performed according to Dai et al. (2015).

β-1,3-glucanase activity was assayed using *Laminaria digitata* laminarin (Sigma) as the substrate. Approximately 0.2 g of fresh fibers were homogenized in 5 ml of 50 μmol of Na-acetate buffer (pH 5.0) with a chilled mortar and pestle. The homogenate was centrifuged at 15,000 × g for 15 min. The supernatant was used directly for enzyme assays. The assay mixture contained 0.4 ml of 1 mg/ml laminarin resolved in 50 μmol of Na-acetate buffer (pH 5.0) and 0.01 ml of an enzyme solution in a total volume of 0.41 ml. After incubation at 37°C for 15 min, the amount of released reducing sugars was determined by the Somogyi-Nelson method (Somogyi, 1952; Nelson, 1957). One unit of activity was defined as the amount of enzyme needed to catalyze the release of reducing sugar groups equivalent to 1 nmol of glucose per second, and specific activity was expressed as units per gram of fiber fresh weight.

### The assessment of gene expression by RT-PCR

Total RNA from fiber tissues was extracted according to Ruan et al. (1997). First-strand cDNA was synthesized from 5 μg of total RNA using a RevertAid™ Premium first strand cDNA synthesis kit (Fermentas). PCR for the genes *SuSy, SPS, invertase*, β-*1,3-glucanase*, β-*1,4-glucanase, expansin*, and *XET* was performed using cDNA in a 20-μL reaction volume with the amplification conditions provided in Table 2. The amplification products were electrophoresed on a 1.0% agarose gel at 100 V in TBE buffer (0.4 M Tris-borate, 0.001 M EDTA, pH 8.0) using DNA ladders of known concentration. Gels were stained with ethidium bromide and visualized on a Uvi Pro Gel Documentation System (GDS-8000, Cambridge, UK). PCR amplification of *18S* mRNA was performed to permit normalization between treated and control samples. Expression analysis of each gene was confirmed by 3 independent reactions using forward and reverse primers specific to each gene. The gel images of each gene can be seen in the Supplementary Material (Figure S1). Relative gene expression was calculated using the following formula:

$$\begin{array}{l} \delta \%=\left(\text{Waterlogging}-\text{W}{\text{L}}_{0}\right)∕\text{W}{\text{L}}_{0}\times 100 \end{array}$$

**Table 2:** **Primers, Tm and Cycles of PCR in the experiment**.

**Gene and accession number**	**Primers**	**Tm (°C)**	**Cycles**	**Length of amplified DNA (bp)**
Expansin (AF043284)	Forward: 5′-AGTCGAACCATAACCGTGACAGCC-3′ reverse: 5′-CCCAATTTCTGGACATAGGTAGCC-3′	56	23	328
β-1,4-glucanase (D88417)	Forward: 5′-GTTCACCACCGAGGTTCTTC-3′ reverse: 5′-TCTTGCCCTTGTGATTCCAG-3′	54	28	415
β-1,3-glucanase (D88416)	Forward: 5′-GAGGACATACAAAGCCTCGCA-3′ reverse: 5′-AGGTTGTAGTATTCCAAGCCT-3′	58	33	455
Invertase (FJ915120)	Forward: 5′-ATCGGGTTGAAAGTGGATTATG-3′ reverse: 5′-TCCGTTGGATACACTCTTGATG-3′	58	30	692
Sucrose synthase (U73588)	Forward: 5′-AGAACCCAAAGTTGCGTGAG-3′ reverse: 5′-ACCGTTACAGGTTGCGAATG-3′	58	30	305
Sucrose phosphate synthase (DQ6773345)	Forward: 5′-ACAA TG CCAGGAGTTTATCG-3′ reverse: 5′-GTCACCAGCATCTGC GTAAT-3′	55	30	345
Endoxyloglucan transferase (*XET*) (AY189971)	Forward: 5′-GATTTTATCTTGTGTTGTTACACTTTC-3′ reverse: 5′-GATATTGGTTTGAACCGTATATGGC-3′	53	36	356
18 S rRNA (L24145)	Forward: 5′-AATCCCTTAACGAGGATCCATT-3′ reverse: 5′-GGCATCGTTTATGGTTGAGACT-3′	55	34	507

### Weather data

Weather data for Nanjing were collected from the National Meteorological Information Center located at Nanjing Agricultural University close to the experimental station. The monthly average weather data consisting of the mean daily temperature (MDT), mean daily maximum temperature (MDT_max_), mean daily minimum temperature (MDT_min_), and total heat units from May to October during the cotton growth period for the 2 years of this study are given in Table 3. Total heat units were calculated using the formula:

∑[(maximum temperature +minimum temperature)/2−15°C]

**Table 3:** **Air temperatures, total duration of sunshine and rainfall, mean relative humidity during the experiments (May–October) in 2011 and 2012**.

**Year**	**Month**	**MDT^a^ (°C)**	**MDT_min_ (°C)**	**MDT_max_ (°C)**	**Heat units (°C -days)**	**Total duration of sunshine (h)**	**Mean relative humidity (%)**	**Total rainfall (mm)**
Long term average (1995–2010)		23.0	19.4	27.5	261	123	70	129
2011	May	22.3	17.4	28.1	225	232	57	41
	June	24.7	21.4	29.1	289	129	77	313
	July	28.1	25.3	31.8	404	144	79	278
	August	27.0	24.4	30.9	375	137	80	284
	September	23.0	20.0	27.1	246	157	71	13
	October	17.6	14.3	21.9	81	155	69	29
	Average	23.8	20.5	28.2	270	159	72	160
2012	May	21.9	17.5	26.9	208	198	66	62
	June	25.5	22.1	29.6	312	142	69	18
	July	29.4	25.7	33.8	441	245	68	176
	August	28.1	25.1	32.2	407	198	72	198
	September	22.3	19.0	26.5	217	184	71	69
	October	18.3	14.4	23.2	103	184	64	55
	Average	24.2	20.6	28.7	281	192	68	96

a *MDT, MDT_min_, MDT_max_ stand for mean daily temperature, mean daily minimum temperature and mean daily maximum temperature*.

### Statistical analysis

Three-way analysis of variance (ANOVA) was performed. Growing season, blocks, and block interactions were included as random effects. Waterlogging time and fruiting branches were included as fixed effects. Significant differences in means between the treatments were compared by the protected least significant difference (LSD) procedure at *P* < 0.05. ANOVA and the LSD test were conducted using the SPSS 17.0 software program (SPSS Inc., 2008). Figures were prepared using the Origin 9.0 software program.

## Results

### Weather data during cotton growth seasons

Weather data during cotton growth seasons in the 2 years differed (Table 3). The temperature, heat units and sunshine hours were higher while rainfall was lower in 2012 compared to 2011. Notably, higher MDT and MDT_max_ occurred in July and August of 2012.

### Fiber quality

Fiber length and strength were significantly influenced by waterlogging (Table 4). The fiber length at each fruiting branch was significantly reduced in cotton after waterlogging for 6, 9, and 12 days. The coefficient of variation showed that the fiber length at FB_6–7_ was most sensitive to waterlogging during flowering and boll formation, but was the least sensitive for fibers at FB_14–15_, with the fiber lengths at WL_9_ and WL_12_ markedly lower than that at WL_0_. Obvious decreases in the fiber strength at FB_6–7_ and FB_10–11_ were observed for cotton after waterlogging for 6, 9, and 12 days, and the strength decreased with the increased duration of waterlogging. An opposing trend was observed for the fiber strength at FB_14–15_ after waterlogging, with the maximum value observed at WL_6_.

**Table 4:** **Effect of the waterlogging treatment on fiber length and strength at different main-stem fruiting branches in 2011 and 2012**.

**Fruiting branches**	**Waterlogging treatment**	**UHML**^b^ **(mm)**		**Strength (cN/tex)**	
		**2011**	**2012**	**2011**	**2012**
FB_6–7_	WL_0_^a^	31.5a^c^	31.1a	30.2a	30.6a
	WL_3_	30.8b	30.4a	29.6b	29.8b
	WL_6_	30.3c	29.2b	28.9c	29.2b
	WL_9_	29.4d	28.6bc	28.3d	28.2c
	WL_12_	28.3e	28.1c	27.7d	27.8c
FB_10–11_	WL_0_	30.9a	31.8a	31.1a	30.2a
	WL_3_	30.7ab	31.6a	30.7b	29.4ab
	WL_6_	30.4bc	31.2a	30.4b	29.5ab
	WL_9_	30.2cd	30.3b	30.1c	28.7b
	WL_12_	29.8d	29.3c	29.9c	27.8c
FB_14–15_	WL_0_	31.6a	31.2a	28.6d	30.0b
	WL_3_	31.1b	31.0a	29.3bc	30.6ab
	WL_6_	30.9b	29.7a	29.7a	30.9a
	WL_9_	30.4c	29.8a	29.1c	30.5ab
	WL_12_	30.2c	29.7a	29.5ab	30.2ab
Year (Y)		8.50^**^^d^		12.06^**^	
Fruiting branches (FB)		20.50^**^		48.92^**^	
Waterlogging (WL)		80.73^**^		92.10^**^	
Y × FB		7.70^**^		74.78^**^	
Y × WL		1.11		1.92	
FB × WL		2.72^**^		18.79^**^	
Y × FB × WL		1.49		1.13	

a *WL_0_, WL_3_, WL_6_, WL_9_, and WL_12_ stand for waterlogging days of 0, 3, 6, 9, and 12 days respectively*.

b *UHML was short for upper half mean length*.

c *Values followed by different letters within the same column are significantly different at P = 0.05 probability level. Each data represents the mean of three replications*.

d *NS, not significant*;

*, ** *Significantly different at the 0.05 and 0.01 probability levels*.

### Cellulose, sucrose and β-1,3-glucan content in cotton fiber

**Figure 1:**
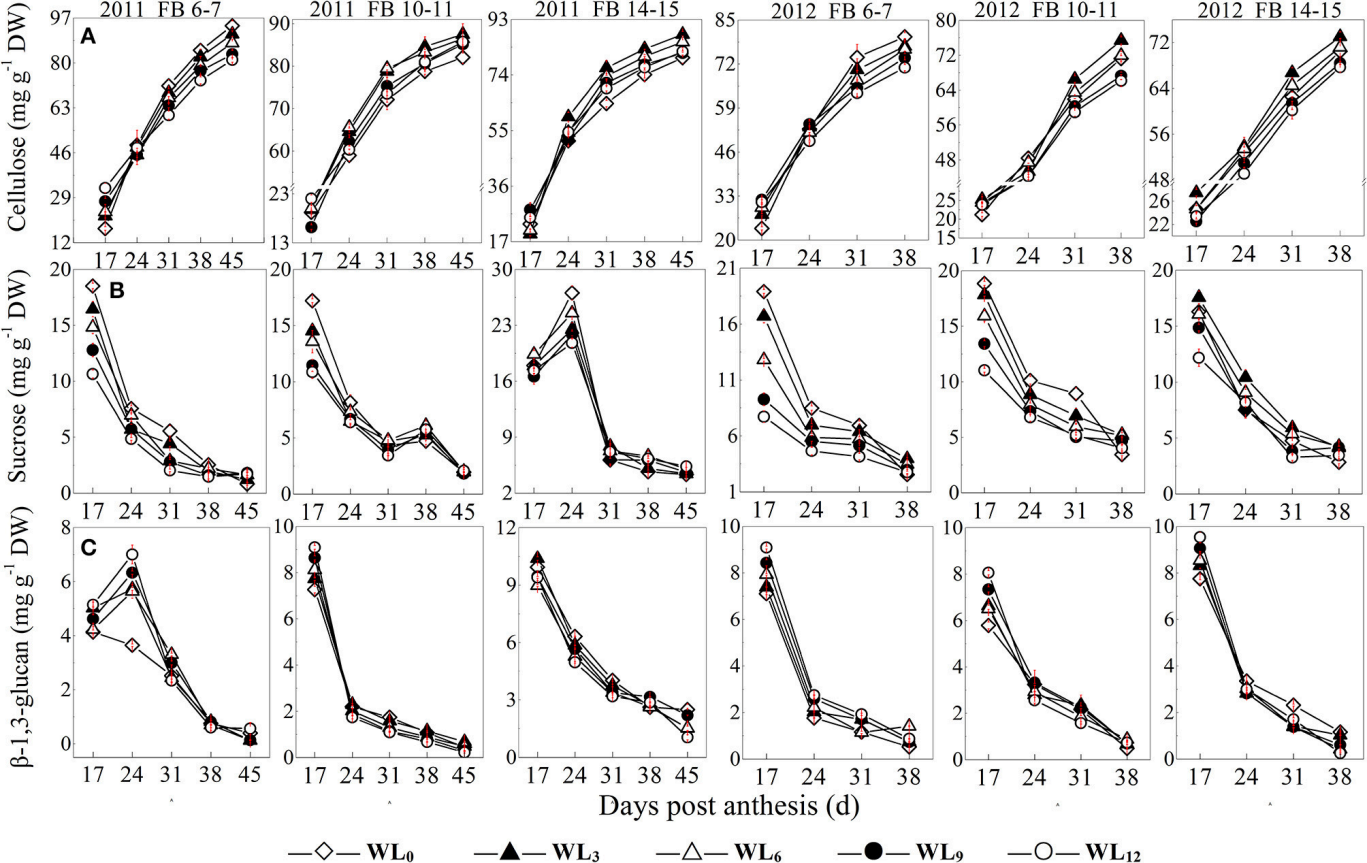
**Effects of the waterlogging time on dynamic changes in fiber cellulose (A), sucrose (B), and β-1,3-glucan (C) content at different main-stem fruiting branches**. Open diamond, closed triangle, open triangle, closed circle and open circle represent WL_0_, WL_3_, WL_6_, WL_9_, and WL_12_ (waterlogging for 0, 3, 6, 9, and 12 days), respectively.

The sucrose content at FB_6–7_ and FB_10–11_ was decreased after waterlogging, whereas a slight increase was observed at FB_14–15_ at WL_3_ and WL_6_. A much more significant reduction in the fiber sucrose content was observed for FB_6–7_. Little difference was found in the fiber cellulose content between WL_3_ and WL_0_, whereas WL_6_, WL_9_, and WL_12_ exhibited a significantly reduced fiber cellulose content at FB_6–7_, which tended to decrease with increasing waterlogging days. The fiber cellulose contents were increased at WL_3_ and WL_6_ for FB_10–11_ and FB_14–15_ compared to the content at WL_0_. The pattern of the fiber cellulose content at WL_9_ and WL_12_ differed between the 2 years. In 2011, no significant difference was observed among WL_9_, WL_12_, and WL_0_, whereas the fiber cellulose content was lower for WL_0_ compared to WL_9_ and WL_12_ in 2012. The pattern of the fiber β-1,3-glucan content after waterlogging was different at each fruiting branch. The fiber β-1,3-glucan content at FB_6–7_ was significantly increased after waterlogging, with the maximum content observed at WL_12_. An obvious reduction in the fiber β-1,3-glucan content was found at FB_10–11_ and FB_14–15_ at 24 DPA after waterlogging (Figure 1).

### Changes in sucrose degradative enzyme activity

**Figure 2:**
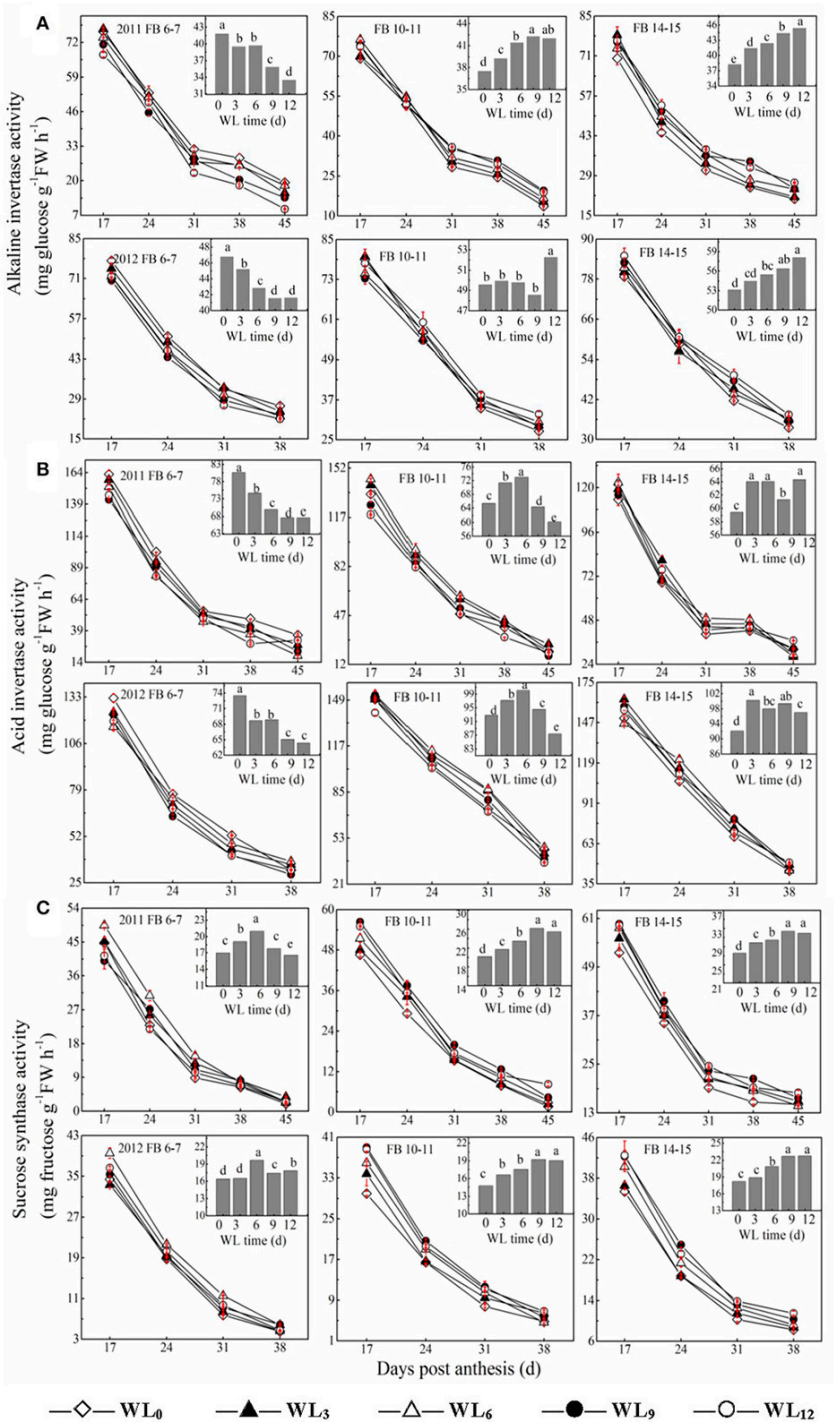
**Effects of the waterlogging time on dynamic changes in fiber sucrose degradative enzymes activities at different main-stem fruiting branches (A, alkaline invertase B, acid invertase; and C, sucrose synthase)**. Columns represent the average activities per day post-anthesis. Data (*n* = 3) are represented as the mean values ± SD calculated from three replicates. Open diamond, closed triangle, open triangle, closed circle and open circle represent WL_0_, WL_3_, WL_6_, WL_9_, and WL_12_ (waterlogging for 0, 3, 6, 9, and 12 days), respectively. WL indicates waterlogging.

The activity of acid invertase was higher than the alkaline invertase activity and decreased with fiber development. Changes in acid and alkaline invertase activities were similar across all of the tested cotton fibers (Figures 2A,B). The average fiber acid invertase activity between the years decreased by 7.0–14.3% for WL_3–12_ at FB_6–7_. Differences were observed in acid invertase activity among waterlogging treatments at FB_10–11_ and FB_14–15_. WL_3–9_ led to 6.9–0.1% and 4.2–1.6% increases in fiber acid invertase activity at FB_10–11_ and FB_14–15_, respectively. A decrease in fiber alkaline invertase activity was observed at FB_6–7_, whereas the opposite trend was found at FB_10–11_ and FB_14–15_. The average fiber alkaline invertase activities were reduced by 4.5–15.5% under WL_3–12_ at FB_6–7_, but were increased by 2.7–8.7% and 5.4–14.0% at FB_10–11_ and FB_14–15_.

SuSy activity in cotton fibers decreased with fiber development (Figure 2C). Waterlogging enhanced fiber SuSy activity at each fruiting branch. The highest value at FB_6–7_ was reached under WL_6_, which reflected an increase of 26.9% compared to WL_0_, whereas the minimum activity among waterlogging treatments was observed for WL_12_, which was reflective of a 9.9% increase compared to WL_0_. The average fiber SuSy activities increased by 10.0–30.0% and 6.3–21.2% under WL_3–12_ at FB_10–11_ and FB_14–15_, respectively, with the maximum activity observed at WL_9_ and WL_12_.

### Changes in sucrose synthesis enzyme activity

**Figure 3:**
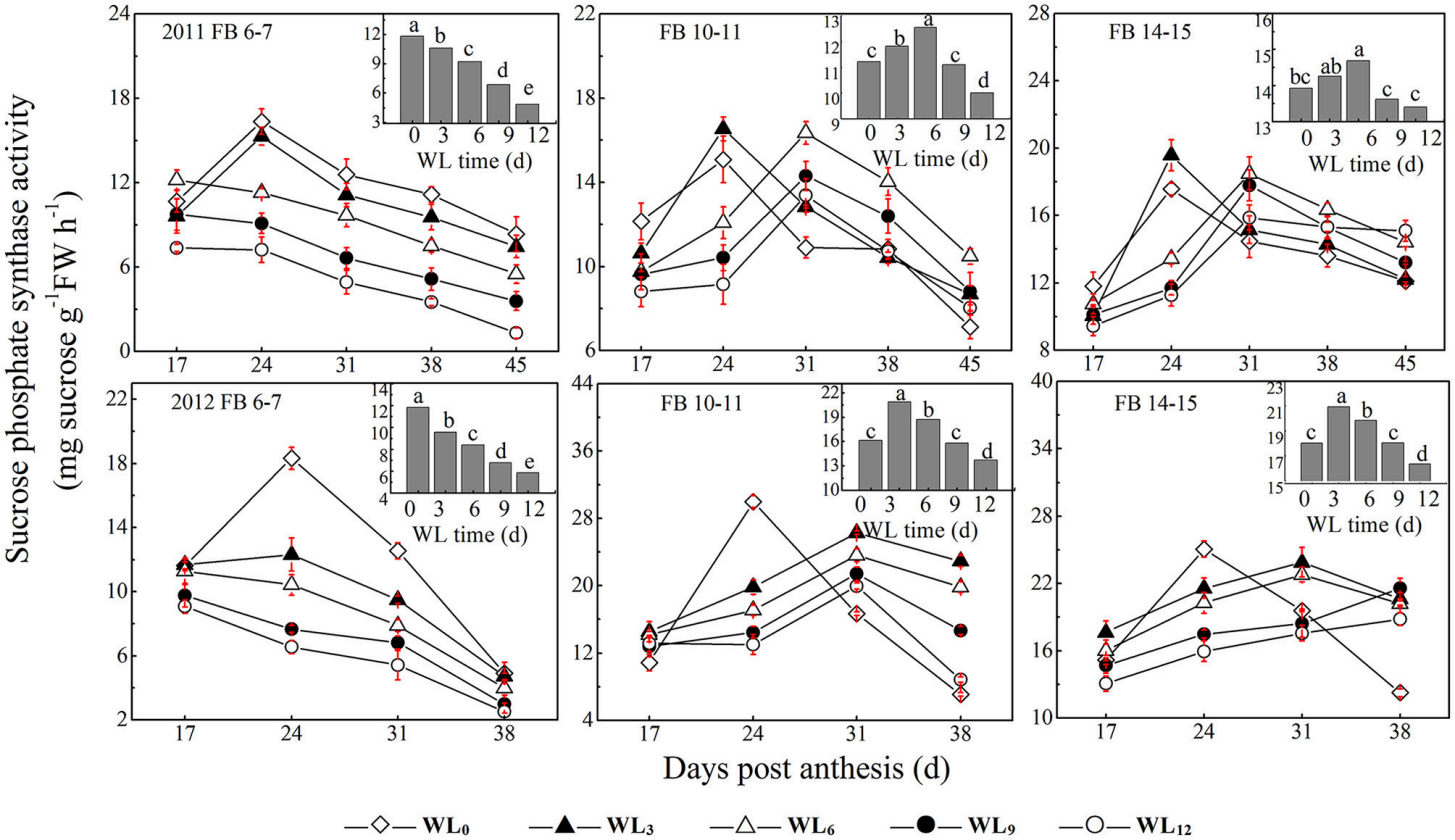
**Effects of the waterlogging time on dynamic changes in fiber sucrose-synthesis enzyme activity at different main-stem fruiting branches (sucrose phosphate synthase)**. Columns represent the average activities per day post-anthesis. Data (*n* = 3) are represented as the mean values ± SD calculated from three replicates. Open diamond, closed triangle, open triangle, closed circle and open circle represent WL_0_, WL_3_, WL_6_, WL_9_, and WL_12_ (waterlogging for 0, 3, 6, 9, and 12 days), respectively. WL indicates waterlogging.

SPS activity increased from 17 DPA until it peaked and then declined significantly until the boll opening date. The patterns of fiber SPS activity after waterlogging were dependent on the fruiting branch (Figure 3). The fiber SPS activities were reduced at FB_6–7_ and peaked early after waterlogging, while a different pattern was observed at FB_10–11_ and FB_14–15_. The average SPS activities were increased by 17.4–13.8% and 9.3–7.8% under WL_3–6_, whereas they were decreased by 12.8 and 6.5% under WL_12_ at FB_10–11_ and FB_14–15_, respectively.

### Changes in β-1,3-glucanase activity

**Figure 4:**
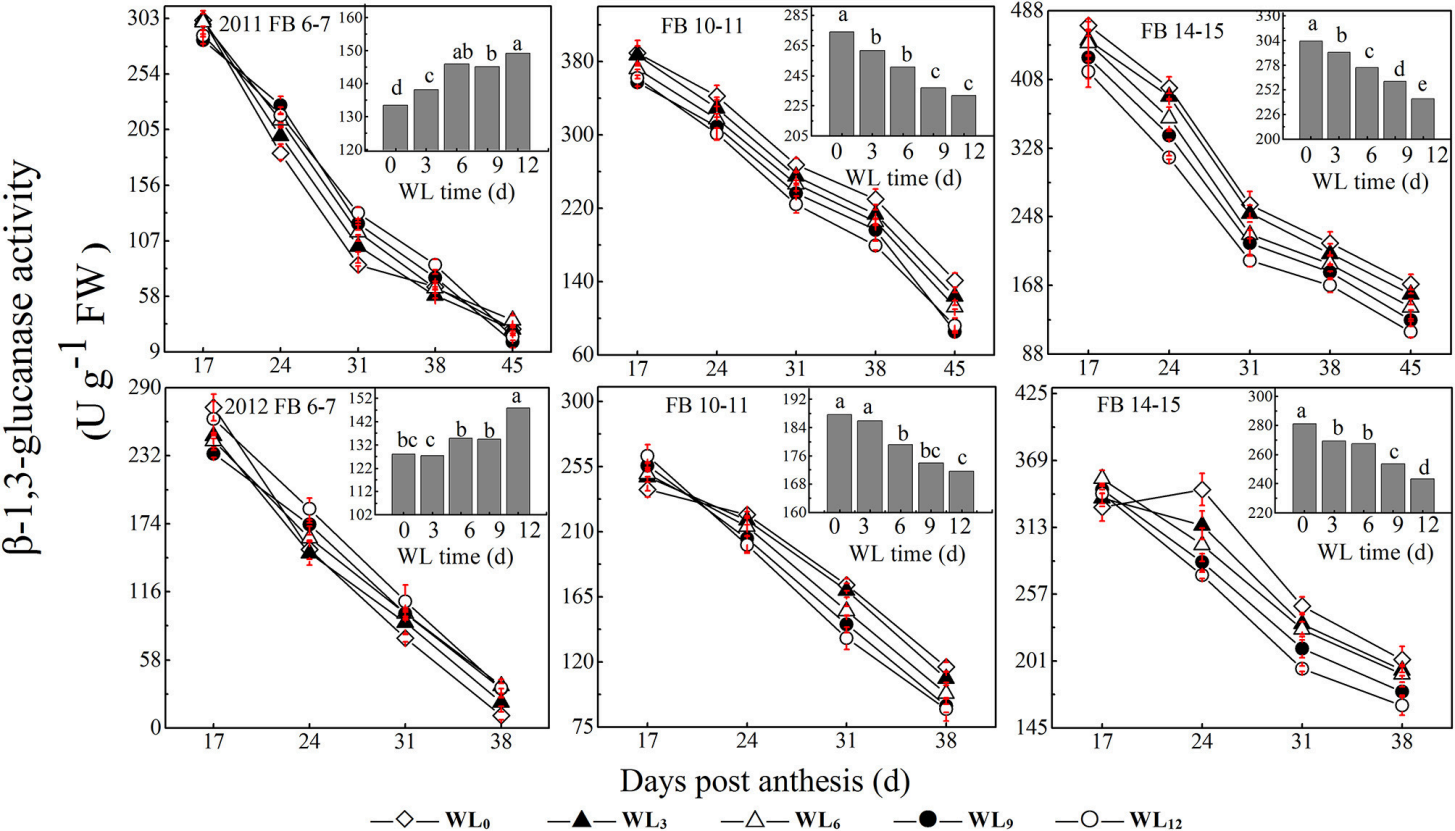
**Effects of the waterlogging time on dynamic changes in fiber β-1,3-glucanase activity at different main-stem fruiting branches**. Columns represent the average activities per day post-anthesis. Data (*n* = 3) are represented as the mean values ± SD calculated from three replicates. Open diamond, closed triangle, open triangle, closed circle and open circle represent WL_0_, WL_3_, WL_6_, WL_9_, and WL_12_ (waterlogging for 0, 3, 6, 9, and 12 days), respectively. WL indicates waterlogging.

The change in β-1,3-glucanase activity after waterlogging opposed that of alkaline invertase. The average fiber β-1,3-glucanase activities increased by 11.8–4.5% and 1.5–13.7% under WL_3–12_, with the maximum value observed under WL_6−9_ and WL_12_ at FB_6−7_, respectively. Moreover, the average fiber β-1,3-glucanase activities were reduced with waterlogging time by 2.7–12.0% and 4.1–16.8% at FB_10–11_ and FB_14–15_ (Figure 4), respectively. The coefficient of variation reveals that SPS was the most sensitive of the enzymes to waterlogging at each fruiting branch, indicating a strong inhibition of sucrose synthesis. SuSy was the second most sensitive enzyme to waterlogging, indicating that SuSy was the main enzyme responsible for catalyzing sucrose breakdown in the fibers after waterlogging. Moreover, 24–45 DPA during fiber development was much more influenced after waterlogging.

### Gene expression during cotton fiber development

**Figure 5:**
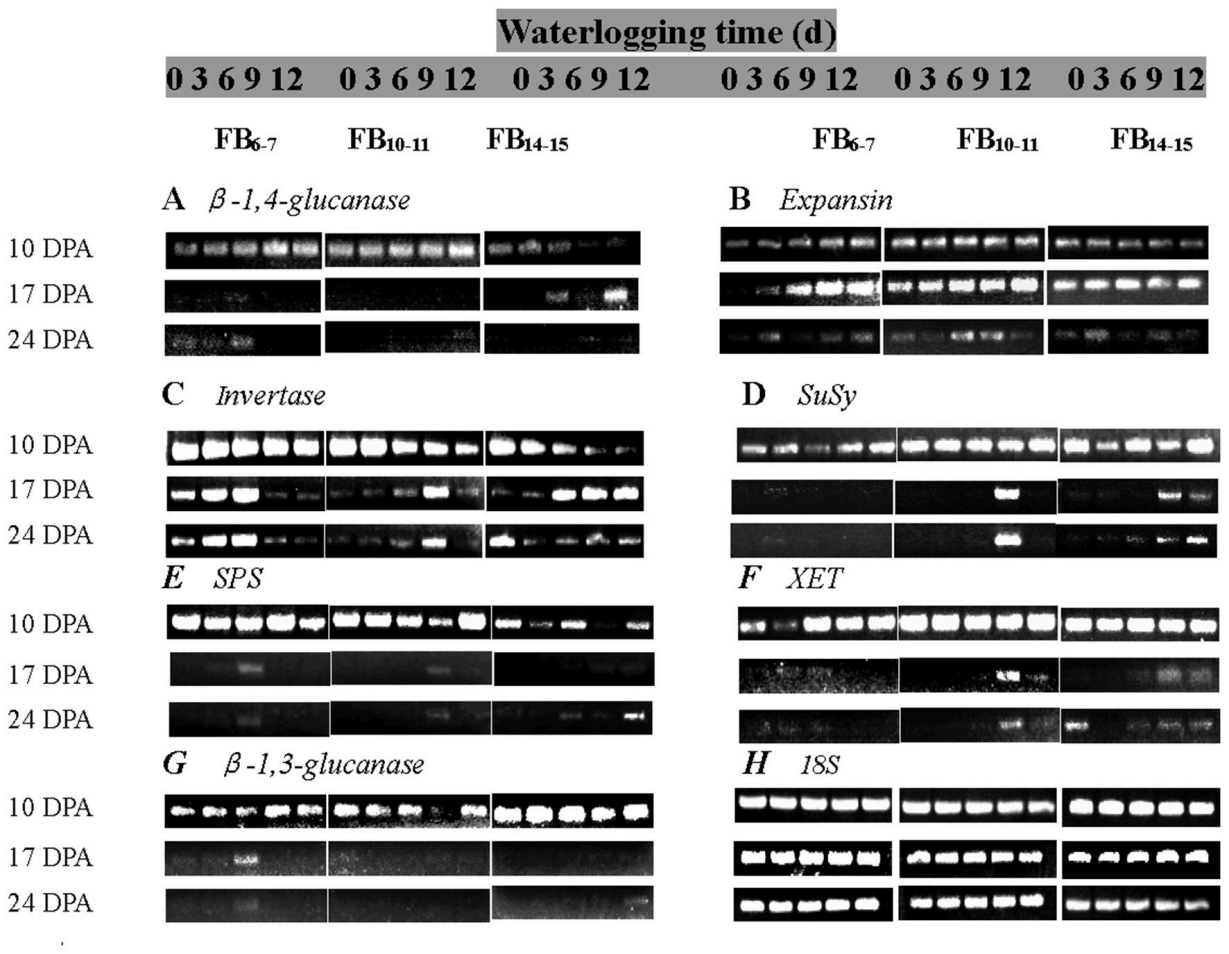
**Effects of the waterlogging time on gene expression in fibers at different main-stem fruiting branches (A, β-*1,4-glucanase*; B, *Expansin*; C, *Invertase*; D, *SuSy*; E, *SPS*; F, *XET*; G, β-*1,3-glucanase*)**. RT-PCR was repeated three times and representative results are shown. **(H)** 18S was amplified as a positive control.

β-*1,4-glucanase* mRNA was most abundantly expressed in the early stage of fiber development at 10 DPA and rapidly decreased thereafter and was also significantly affected by waterlogging (Figure 5A). A distinctive pattern was observed for the β-*1,4-glucanase* expression level in fibers at each fruiting branch, which was very low at FB_14–15_, but higher for fibers at FB_6–7_ and FB_10–11_. The total expression of fiber β-*1,4-glucanase* was increased by approximately 0.79–1.97, 0.65–3.47, and 4.43–30.7-fold under WL_3–12_ compared to that of WL_0_ at FB_6–7_, FB_10–11_ and FB_14–15_, respectively.

RT-PCR analysis of the *expansin* gene showed that the *expansin* transcript level increased from 10 DPA and reached maximal levels at 17 DPA, then decreased thereafter. The fiber *expansin* genes were found to be significantly induced after waterlogging at FB_6–7_ and FB_10–11_, with increases in the transcript level of 4.04 to 10.37- and 2.58 to 7.90-fold compared to that of WL_0_(Figure 5B).

Under the control condition WL_0_, the *invertase* mRNA level decreased with fiber development (10–24 DPA). Significant down-regulation of the fiber *invertase* level was observed at 10 DPA for each fruiting branch. WL_3_ and WL_6_ increased the expression of *invertase*, whereas it was inhibited at 17 DPA and 24 DPA under WL_9_ and WL_12_ at FB_6–7_. The transcript levels of fiber *invertase* at FB_10–11_ and FB_14–15_were up-regulated under waterlogged cottons, and the invertase level increased with waterlogging days (Figure 5C), consistent with the changes of fiber *invertase* activities.

Fiber *SuSy* was highly expressed at 10 DPA and rapidly decreased thereafter. Its expression was obviously enhanced by waterlogging at each fruiting branch. Analysis of the total *SuSy* expression level showed that it had increased by 0.72–3.63, 1.24–9.43, and 5.52–27.72-fold under waterlogging conditions at FB_6–7_, FB_10–11_ and FB_14–15_, respectively, compared to the level at WL_0_ (Figure 5D).

The pattern of fiber *SPS* expression was similar at each fruiting branch. The duration of *SPS* was prolonged, whereas the expression levels appeared to be irregular after waterlogging (Figure 5E)_._

Changes in the expression level of *XET* in cotton fibers were similar to those of *SuSy*. Analysis of the total expression of *XET* revealed that it had increased by 5.53–0.62, 1.63–13.28, and 9.24–6.97-fold under WL_3–12_ at FB_6–7_, FB_10–11_ and FB_14–15_, respectively (Figure 5F).

The mRNA levels of β-*1,3-glucanase* reached maximal levels at 10 DPA and decreased thereafter. Waterlogging up-regulated the level of fiber β-*1,3-glucanase* expression at FB_6−7_, and the maximum level was reached at WL_9_. A significant reduction in the total mRNA levels of β-*1,3-glucanase* was observed at FB_10−11_ and FB_14–15_ after waterlogging (Figure 5G).

## Discussion

Events in the soil, including O_2_ deficiency, result from waterlogging in the root zone and change the redox status of nutrients, making them unavailable (e.g., nitrogen) or potentially toxic for plants. Root-derived hormones that are transported in the xylem have long been associated with oxygen deficits. These below-ground effects (i.e., impaired root growth, nutrient uptake and transport, hormonal signaling) affect the shoots, interfering with canopy development, photosynthesis and radiation use efficiency, which ultimately affects the yield (Kuai et al., 2014, 2015) and fiber quality. Differences in environmental conditions among years had potential to alter plant development. In the two experimental years, the average MDT, MDTmax, MDTmin, total duration of sunshine and rainfall from April to October were near the long-term average. For the boll development period of July to October, the long-term average of MDT from 1995 to 2010 was 16.9–29.9°C. Mean daily temperature for the 2 years was 17.6–28.1°C, 18.3–29.4°C, respectively, near the long-term average, above or below 1°C (Table 3). This indicated that the climatic conditions in the 2 years were similar and normal. The 2011 and 2012 growing seasons could be considered typical for the Yangzi River Region. However, the contrasting weather conditions in the 2 years of our study apparently caused a difference in fiber growth between 2011 and 2012. Higher temperature and abundant sunshine in 2012 during the growing period (June–August) resulted in shorter boll maturity period as shown by Kuai et al. (2014). Based on the experiments in 2011 and 2012, sucrose metabolism and cellulose synthesis were both significantly affected in the after-waterlogging period, thus resulting in changes in fiber length and strength. Significant difference was observed in years (Table 4), indicating that the effects of waterlogging on fiber development depended on weather conditions during waterlogging and fiber growth season. Waterlogging accompanied by higher temperature damaged more fruiting buds (squares) and caused a larger amount of square shed. This situation meant fewer available blooms and altered the source-sink relationship of the remaining fruit (Kuai et al., 2015), which resulted in difference in fiber development.

As shown previously, cotton fiber length and strength are significantly affected by waterlogging and are directly regulated by sucrose metabolism (Shu et al., 2009; Dai et al., 2015). Because sucrose synthase and invertase are both involved in sucrose cleavage in sink tissues, their activities are regarded as biochemical markers of sink strength. Our results show that the activities of fiber acid invertase and alkaline invertase were restricted after waterlogging, whereas fiber sucrose synthase activity was higher at FB_6−7_, with the maximum level observed under WL_6_ (Figure 2). The up-regulation of the fiber *SuSy* level was observed in waterlogged cotton plants (Figure 5D), in agreement with its activity level (Figure 2). Increased sucrose cleavage by SuSy in response to stress conditions contributed to increased ATP production at constant sucrose consumption, consistent with a report by Guglielminetti, who found that the activity of invertase in rice seedlings decreased whereas the activity and gene expression of SuSy increased under anoxic conditions (Guglielminetti et al., 1995). Consequently, we concluded that SuSy was the main enzyme responsible for sucrose cleavage in cotton fibers at FB_6–7_ after short-term waterlogging. Previous studies have also reported that the generation of phosphorylated sugars from sucrose via SuSy is an energetically favorable means of sustaining glycolysis (Huang et al., 2008) and supporting sucrose metabolism during post-stress recovery (Santaniello et al., 2014). However, the increase in SuSy activity could not compensate for the decrease in the activities of fiber acid and alkaline invertase at those fruiting branches, which finally led to the reduction in the ability to break down sucrose, indicating severe inhibition of sucrose cleavage metabolism after waterlogging. This resulted in a significant reduction in the fiber sucrose content at FB_6–7_ (Figure 1).

**Figure 6:**
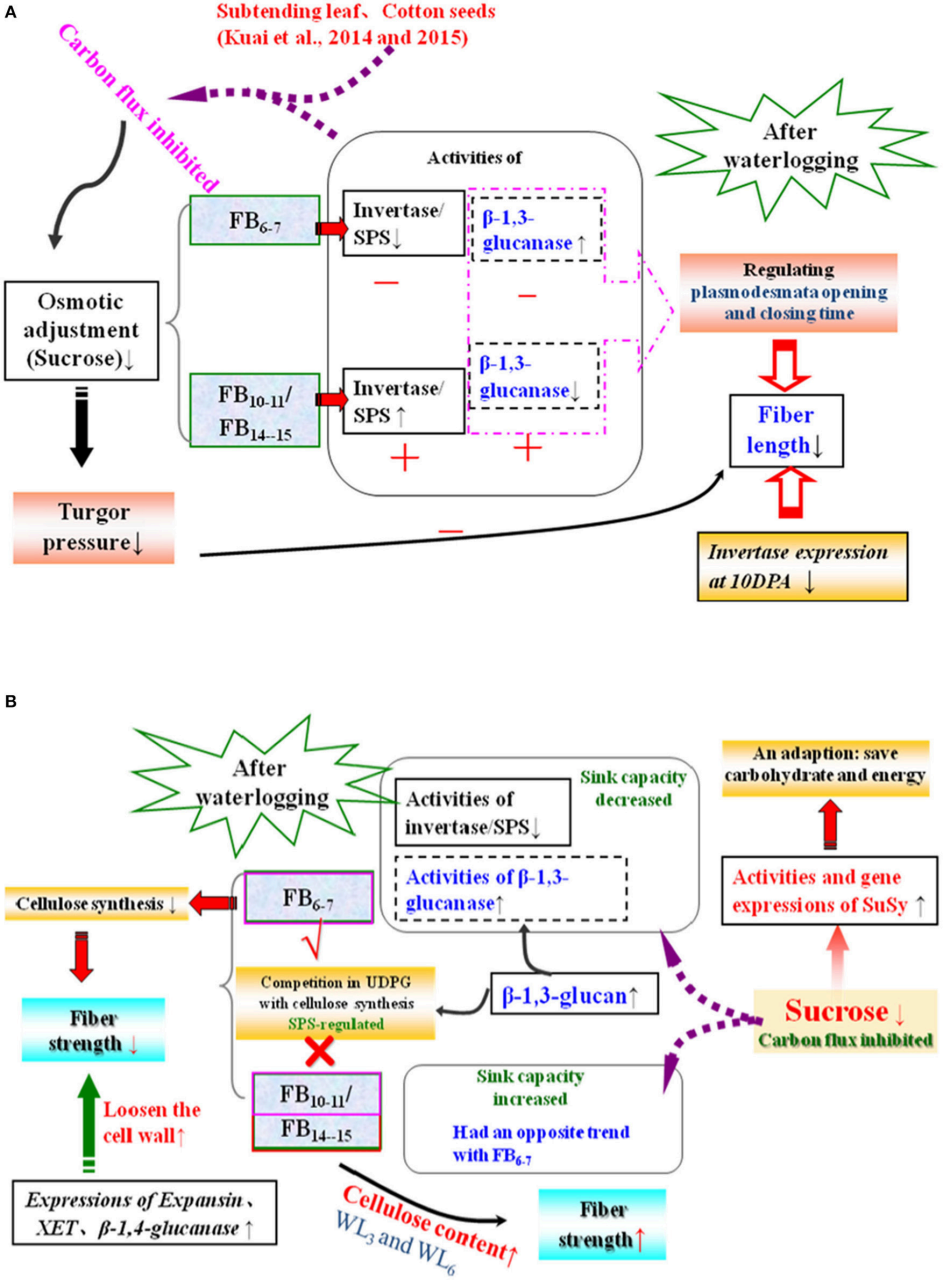
**Simplified metabolic scheme and changes in fiber length (A) and strength (B) after waterlogging**. The reactions analyzed in the present study involving the metabolism of sugars are shown. Enzymes and metabolites that increased or decreased after waterlogging with respect to cotton bolls of the same age are indicated by “+,” “↑” and “−” “↓,” respectively, while “√” and “×” represent the existence and disappearance of competition, respectively. FB, fruit branches; SuSy, sucrose synthase; SPS, sucrose phosphate synthase; XET, endoxyloglucan transferase; WL_3_, waterlogging for 3 days; WL_6_, waterlogging for 6 days.

It has been hypothesized that high levels of enzymes involved in the breakdown of sucrose in the sink would increase sink capacity by lowering the local concentration of sucrose, thereby generating a gradient that permits further unloading of sucrose from the phloem (Ranwala and Miller, 1998). Thus, decreased inhibition of sucrose cleavage resulted in less sucrose translocation from the source to the sink at FB_6–7_. Recovery after waterlogging may be limited by a deficiency of the carbohydrates used for the regeneration of roots and shoots. When cotton plants are re-aerated after drainage, their primary axes initiate lateral roots after the death of the apical meristem. Increased demand for carbohydrates during root formation may lead to a reduction in the translocated carbohydrates available for sucrose biosynthesis in the developing fruit (Najeeb et al., 2015). This was another reason for the reduction in the fiber sucrose content after waterlogging. However, due to the indeterminate growth habits of cotton, fibers of the relatively new fruiting branches FB_10–15_ exhibited a degree of compensation. The activities of fiber alkaline invertase, acid invertase and SuSy all increased in waterlogged cottons compared to those of WL_0_, with the best performance observed in WL_12_, WL_6_ and WL_12_ (Figure 2). These results indicate an increase in sink strength in the fibers at FB_10–15_ after waterlogging and that acid invertase was more sensitive to waterlogging than alkaline invertase. Similar results were found by Liang et al. (2001). However, the fiber sucrose content of these fruiting branches remained lower than that of unwaterlogged cottons, with the exception of WL_3_ and WL_6_ at FB_14–15_ (Figure 1B), indicating that waterlogging for more than 6 days led to an irreversible limitation in sucrose translocation to fibers, as indicated by the fact that photosynthesis in the leaves subtending the cotton boll was restricted (Kuai et al., 2014). The fiber cells expand rapidly through the concerted action of turgor pressure and cell wall relaxation. Compelling evidence has indicated a major role for osmotically active solutes in fiber elongation through the generation of cell turgor, including soluble sugars, K^+^ and malic acid, with sucrose being the major component of soluble sugar (Ruan et al., 1997). The fiber sucrose content exhibited a similar trend to the fiber length (Figure 1B; Table 4), indicating that lower cell turgor caused by reduced sucrose content resulted in a shorter fiber length after waterlogging (Figure 6A). Previous work has shown that high invertase activity is required for cotton fiber elongation through osmosis. Fibers exhibiting higher invertase activity had faster elongation rates (Wang et al., 2010), whereas the over-expression of an Arabidopsis sucrose phosphate synthase gene resulted in substantially elevated concentrations of sink sucrose pools, which was ascribed to internode elongation as well as an increased stem diameter and fiber length compared to wild-type plants (Park et al., 2008). The mRNA levels of fiber invertase at 10 DPA (a critical period for fiber elongation) were down-regulated at each fruiting branch after waterlogging (Figure 5C), in agreement with Narsai et al. (2011), suggesting that the inhibition of *invertase* expression at 10 DPA after waterlogging was responsible for the decreased fiber length after waterlogging (Figure 6A).

A decrease in the sucrose content and sucrose cleavage led to a shortage of UDPG, resulting in reduced cellulose deposition under WL_6_, WL_9_ and WL_12_ at FB_6–7_. The fiber cellulose content was slightly increased under WL_3_ and WL_6_, whereas it remained lower than WL_0_ under WL_9_ and WL_12_ at FB_10–15_ (Figure 1A). The change in fiber cellulose exhibited a similar trend to that of fiber strength. Fiber β-1,3-glucanase activities were elevated at FB_6–7_ (Figure 4), accompanied by increased β-1,3-glucan levels (Figure 1C), which was consistent with a report that an excess of β-1,3-glucan was deposited in the roots of anoxic maize seedlings (Subbaiah and Sachs, 2001). An opposite trend was observed at FB_10–15_. In addition, changes in the expression of the fiber β-1,3-glucanase gene (Figure 5G) at each fruiting branch were in agreement with those of the β-1,3-glucanase activities and β-1,3-glucan content (Figures 1C, 4). Together, these data indicate that waterlogged cotton fibers at FB_6−7_ direct more UDPG to β-1,3-glucan synthesis and that the β-1,3-glucanase level increased to defend against stress. Meanwhile, the fibers at FB_10−15_ seemed to have acclimated to waterlogging stress, and more UDPG was directed toward cellulose synthesis. SPS is an important enzyme that affects fiber strength as is related to secondary cell wall synthesis in cotton fiber (Babb and Haigler, 2000). SPS is also active in other sucrose-synthesizing organs and tissues, including those adapted to cold or drought (Huber and Huber, 1996). Sucrose synthesis in cotton fiber after waterlogging was inhibited, as reflected in the expression of sucrose phosphate synthase (SPS), except for in fibers at FB_10−15_ after WL_3−6_. The change in SPS activities (Figure 3) was in agreement with that of the fiber cellulose content (Figure 1A), while it was opposite that of fiber β-1,3-glucan after waterlogging (Figure 1C). We therefore assumed that SPS was closely related to carbon allocation to β-1,3-glucan synthesis or cellulose synthesis in cotton fibers after waterlogging. High fiber SPS activities promoted cellulose synthesis, whereas lower SPS activities were favorable to the synthesis of β-1,3-glucan. Similar results have been reported for water-stressed potato tubers (Geigenberger et al., 1999).

The inhibition of cellulose synthesis due to decreased sucrose was responsible for the fiber strength reduction at FB_6–7_ and FB_10–11_ after waterlogging. For fibers at FB_14–15_, the boll period elongated after waterlogging (Kuai et al., 2014), and more cellulose was stockpiled in the fibers of waterlogged cotton, resulting in an increase in fiber strength at FB_14–15_, with the maximum strength observed in WL_6_. During expansion and cell wall reconstruction during fiber development, *expansin*, β-*1,4-glucanase* and *endoxyloglucan transferase* (*XET*) play critical roles in mediating cell wall extensibility via loosening the cell wall (Shimizu et al., 1997). The average mRNA levels of β-*1,4-glucanase, expansin* and *XET* were consistently up-regulated in fibers at each fruiting branch (Figures 5A,B,F), thereby accelerating cell wall loosening and reconstruction and adversely affecting increases in fiber strength at the level of gene expression (Figure 6B). Previous research in maize revealed that hypoxia induces *XET* mRNA expression and protein synthesis (Dennis et al., 2000). Moreover, *XET* is thought to be important in regulating programmed cell death (PCD) in plants (Schünmann et al., 1997). Furthermore, short-term plant survival does not require cellulose synthesis, which supports the formation of new tissues and organs during growth (e.g., leaf, root) due to the high carbon demand of cellulose synthesis (Brown et al., 1996). Therefore, up-regulation of *expansin*, β-*1,4-glucanase* and *XET* expression might be correlated with plant cell PCD after waterlogging stress such that carbon is directed toward basic metabolism to support short-term survival rather than unnecessary growth or evolutionary success. This is in contrast to the results of Albrecht and Mustroph, who found that increased cell wall thickening following cellulose deposition in wheat roots under hypoxia, possibly because the roots are essential organs for plant survival (Albrecht and Mustroph, 2003a). In the present study, we identified the mechanism affecting fiber length and strength after waterlogging with respect to the carbohydrate content, relevant enzymes, and genes expression levels. However, nearly all of the genes examined are members of multigene families and could have different functions during fiber development. To be confident in the changes in expression and to identify the responses to waterlogging of each member in the gene family, quantitative RT-PCR should be performed in a future study.

## Conclusions

The sucrose and cellulose contents of fibers at FB_6–7_ were significantly reduced by a reduction in the activities of SPS and invertase after waterlogging, whereas significant compensation (i.e., the sucrose and cellulose contents increased) was observed in fibers at FB_10–15_ for WL_3–6_. SuSy was the main enzyme responsible for sucrose cleavage in cotton fibers after waterlogging, while SPS was the most sensitive to waterlogging in cotton fibers; decreased SPS activity under waterlogging-stressed conditions induced the synthesis of fiber β-1,3-glucan from sucrose rather than cellulose, and more than 6 days of waterlogging led to an irreversible decrease in its activity. A significant decrease in fiber length under WL_6–12_ was caused by a reduction in the sucrose content and the down-regulation of *invertase* at 10 DPA. Restricted cellulose synthesis and the up-regulation of the fiber *expansin*, β-*1,4-glucanase* and *XET* contents led to markedly decreased fiber strength at FB_6–11_ for WL_6–12_, whereas the fiber strength at FB_14–15_ under WL_3–6_ conditions was enhanced as a result of increased cellulose reserves in the fibers.

## Author contributions

ZZ, YM and BC conceived of the experiments and led the study design. JK and YC carried out the experiments and performed analyses. JK wrote the paper. WZ and YW assisted with study design, data analysis, and writing. All of the authors contributed to editing the manuscript.

### Conflict of interest statement

The authors declare that the research was conducted in the absence of any commercial or financial relationships that could be construed as a potential conflict of interest.

## Supplementary material

Figure S1 **The complete non-cropped image of the gels presented in Figure 5**.

## Abbreviations

CV(%): coefficient of variance
DPA(d): days post anthesis
FB_6–7_, FB_10–11_, FB_14–15_: the sympodial fruiting branch at main-stem nodes 6–7, 10–11, and 14–15
MDT (°C): mean daily temperature
MDTmin (°C): mean daily minimum temperature
MDTmax (°C): mean daily maximum temperature
SPS: sucrose phosphate synthase
SuSy: sucrose synthase
WL_0_, WL_3_, WL_6_, WL_9_, WL_12_: waterlogging days of 0, 3, 6, 9, and 12 days
XET: endoxyloglucan transferase.

## References

[B1] Albrecht G. Mustroph A. (2003a). Localization of sucrose synthase in wheat roots: increased *in situ* activity of sucrose synthase correlates with cell wall thickening by cellulose deposition under hypoxia. Planta 217, 252–260. 10.1023/B:RUPP.0000003280.10924.03 12783333

[B2] Albrecht G. Mustroph A. (2003b). Sucrose utilization via invertase and sucrose synthase with respect to accumulation of cellulose and callose synthesis in wheat roots under oxygen deficiency. Russ. J. Plant Physiol. 50, 813–820. 10.1023/B:RUPP.0000003280.10924.03

[B3] Babb V. Haigler C. (2000). Exploration of a role for sucrose phosphate synthase in cellulose synthesis during secondary cell wall deposition. Abstract 319, in Proceedings of Plant Biology (San Diego, CA: American Society of Plant Physiologists, Rockville, MD), 15–19. Available online at: http://www.aspp.org/annual-meeting/pb-2000/2000.htm

[B4] Bange M. Milroy S. Thongbai P. (2004). Growth and yield of cotton in response to waterlogging. Field Crops Res. 88, 129–142. 10.1016/j.fcr.2003.12.002

[B5] Basra A. S. Malik C. P. (1984). Development of the cotton fiber. Int. Rev. Cytol. 89, 65–113. 10.1016/S0074-7696(08)61300-5

[B6] Brown R. Saxena M. Inder M. (1996). Cellulose biosynthesis in higher plants. Trends Plant Sci. 1, 149–156. 10.1016/S1360-1385(96)80050-1

[B7] Dai Y. Chen B. Meng Y. Zhao W. Zhou Z. Oosterhuis D. M. (2015). Effects of elevated temperature on sucrose metabolism and cellulose synthesis in cotton fibre during secondary cell wall development. Funct. Plant Biol. Plant Biol. 42, 909–919. 10.1071/FP14361 32480733

[B8] Delmer D. P. Haigler C. H. (2002). The regulation of metabolic flux to cellulose, a major sink for carbon in plants. Metab. Eng. 20, 22–28. 10.1006/mben.2001.0206 11800571

[B9] Dennis E. S. Dolferus R. Ellis M. Rahman M. Wu Y. Hoeren F. U.. (2000). Molecular strategies for improving waterlogging tolerance in plants. J. Exp. Bot. 51, 89–97. 10.1093/jexbot/51.342.89 10938799

[B10] Geigenberger P. Reimholz R. Deiting U. Sonnewald U. Stitt M. (1999). Decreased expression of sucrose phosphate synthase strongly inhibits the water stress-induced synthesis of sucrose in growing potato tubers. Plant J. 19, 119–129. 10.1046/j.1365-313X.1999.00506.x 10476059

[B11] Gillham F. E. M. Bell T. M. Arin T. Matthews G. A. Rumeur C. Hearn A. B. (1995). Cotton Production Prospects for the Next Decade. Washington, DC: The World Bank, World Bank Technical Paper.

[B12] Guglielminetti L. Perata P. Alpi A. (1995). Effect of anoxia on carbohydrate metabolism in rice seedlings. Plant Physiol. 108, 735–741. 12228505 10.1104/pp.108.2.735 PMC157395

[B13] Haigler C. H. Ivanova-Datcheva M. Hogan P. S. (2001). Carbon partitioning to cellulose synthesis. Plant Mol. Biol. 47, 29–51. 10.1023/A:1010615027986 11554477

[B14] Harada T. Ishizawa K. (2003). Starch degradation and sucrose metabolism during anaerobic growth of pondweed (*Potamogeton distinctus* A. *Benn.)* turions. Plant Soil 253, 125–135. 10.1023/A:1024585015697

[B15] Hearn A. B. (1995). The principles of cotton water relations and their application in management, in Proceedings of World Cotton Research Conference (1st:1994 Brisbane, Queensland) CSIRO. (Melbourne, VIC).

[B16] Horchani F. Khayati H. Aschi-Smiti S. (2011). Contrasted responses to root hypoxia in tomato fruit at two stages of development. J. Plant Biol. 54, 15–22. 10.1007/s12374-010-9137-4

[B17] Huang S. Colmer T. D. Millar A. H. (2008). Does anoxia tolerance involve altering the energy currency towards PPi? Trends Plant Sci. 13, 221–227. 10.1016/j.tplants.2008.02.007 18439868

[B18] Huber S. C. Huber J. L. (1996). Role and regulation sucrose phosphate synthase in higher plants. Annu. Rev. Plant Physiol. Plant Mol. Biol. 47, 431–445. 10.1146/annurev.arplant.47.1.431 15012296

[B19] Kuai J. Liu Z. Wang Y. Meng Y. Chen B. Zhao W.. (2014). Waterlogging during flowering and boll forming stages affects sucrose metabolism in the leaves subtending the cotton boll and its relationship with boll weight. Plant Sci. 223, 79–98. 10.1016/j.plantsci.2014.03.010 24767118

[B20] Kuai J. Zhou Z. G. Wang Y. H. Chen B. L. Meng Y. L. Zhao W. Q. (2015). The effects of short-term waterlogging on the lint yield and yield components of cotton with respect to boll position. Eur. J. Agron. 67, 61–74. 10.1016/j.eja.2015.03.005

[B21] Liang J. Zhang J. Cao X. (2001). Grain sink strength may be related to the poor grain filling of indica-japonica rice (*Oriza sativa* L.) hybrids. Plant Physiol. 112, 470–477. 10.1034/j.1399-3054.2001.1120403.x 11473706

[B22] Maltby D. Carpita N. C. Montezinos D. Kulow C. Delmer D. P. (1979). β-1,3-Glucan in developing cotton fibers. Plant Physiol. 63, 1158–1164. 10.1104/pp.63.6.1158 16660875 PMC542988

[B23] Najeeb U. Bange M. P. Tan D. K. Y. Atwell B. J. (2015). Consequences of waterlogging in cotton and opportunities for mitigation of yield losses. AoB Plants 7:plv080. 10.1093/aobpla/plv080 26194168 PMC4565423

[B24] Narsai R. Rocha M. Geigenberger P. Whelan J. Dongen J. T. (2011). Comparative analysis between plant species of transcriptional and metabolic responses to hypoxia. New Phytol. 190, 472–487. 10.1111/j.1469-8137.2010.03589.x 21244431

[B25] Nelson M. J. (1957). Colorimetric analysis of sugars. Methods Enzymol. 3, 85–86.

[B26] Park J. Canam T. Kang K. Ellis D. Mansfield S. (2008). Over-expression of an arabidopsis family A sucrose phosphate synthase (SPS) gene alters plant growth and fiber development. Transgenic Res. 17, 181–192. 10.1007/s11248-007-9090-2 17415671

[B27] Pettigrew W. T. (2001). Environmental effects on cotton fiber carbohydrate concentration and quality. Crop Sci. 41, 1108–1113. 10.2135/cropsci2001.4141108x

[B28] Ranwala A. P. Miller W. B. (1998). Sucrose-cleaving enzymes and carbohydrate pool in Lilium longiflorum floral organ. Plant Physiol. 103, 541–550. 10.1034/j.1399-3054.1998.1030413.x

[B29] Ricard B. Rivoal J. Spiteri A. Pradet A. (1991). Anaerobic stress induces the transcription and translation of sucrose synthase in rice. Plant Physiol. 95, 669–674. 10.1104/pp.95.3.669 16668037 PMC1077589

[B30] Ruan Y. L. Chourey P. S. Delmer D. P. Perez-Grau L. (1997). The differential expression of sucrose synthase in relation to diverse patterns of carbon partitioning in developing cotton seed. Plant Physiol. 115, 375–385. 12223814 10.1104/pp.115.2.375 PMC158495

[B31] Ruan Y. L. Jin Y. Yang Y. J. Li G. J. Boyere J. S. (2010). Sugar input, metabolism, and signaling mediated by invertase: roles in development, yield potential, and response to drought and heat. Mol. Plant. 3, 942–955. 10.1093/mp/ssq044 20729475

[B32] Ruan Y. L. Llewellyn D. J. Furbank R. T. (2003). Suppression of sucrose synthase gene expression represses cotton fiber cell initiation, elongation, and seed development. Plant Cell 15, 952–964. 10.1105/tpc.010108 12671090 PMC152341

[B33] Santaniello A. Loreti E. Gonzali S. Novi G. Perata P. (2014). A reassessment of the role of sucrose synthase in the hypoxic sucrose-ethanol transition in Arabidopsis. Plant Cell Environ. 37, 2294–2302. 10.1111/pce.12363 24810896

[B34] Schünmann P. Smith R. Lång V. Matthews P. Chandler P. (1997). Expression of XET-related genes and its relation to elongation in leaves of barley (*Hordeum vulgare* L.). Plant Cell Environ. 20, 1439–1450. 10.1046/j.1365-3040.1997.d01-49.x

[B35] Shimizu Y. Aotsuka S. Hasegawa O. Kawada T. Sakuno T. Sakai F.. (1997). Changes in levels of mRNAs for cell wall-related enzymes in growing cotton fiber cells. Plant Cell physiol. 38, 375–378. 10.1093/oxfordjournals.pcp.a029178 9150611

[B36] Shu H. M. Zhou Z. G. Xu N. Y. Wang Y. H. Zheng M. (2009). Sucrose metabolism in cotton (*Gossypium hirsutum* L.) fibre under low temperature during fibre development. Eur. J. Agron. 31, 61–68. 10.1016/j.eja.2009.03.004

[B37] Somogyi M. (1952). Notes on sugar determination. J. Biol. Chem. 195, 19–23. 14938350

[B38] Su Y. C. Xu L. P. Xue B. T. Wu Q. B. Guo J. L. Wu L. G.. (2013). Molecular cloning and characterization of two pathogenesis-related β-1, 3-glucanase genes ScGluA1 and ScGluD1 from sugarcane infected by Sporisorium scitamineum. Plant Cell Rep. 32, 1503–1519. 10.1007/s00299-013-1463-9 23842883

[B39] Subbaiah C. C. Sachs M. M. (2001). Altered patterns of sucrose synthase phosphorylation and localization precede callose induction and root tip death in anoxic maize seedlings. Plant Physiol. 125, 585–594. 10.1104/pp.125.2.585 11161016 PMC64860

[B40] Triplett B. A. (1993). Using biotechnology to improve cotton fiber quality: progress and perspectives, in Cellulosics: Pulp, Fiber, and Environmental Aspects, Ellis Horwood (Chichester, UK).

[B41] Tucker M. R. Paech N. A. Willemse M. T. Koltunow M. G. (2001). Dynamics of callose deposition and β-1,3-glucanase espression during reproductive events in sexual and apomictic Hieracium. Planta 212, 487–498. 10.1007/s004250000445 11525505

[B42] Updegraff D. M. (1969). Semimicro determination of cellulose inbiological materials. Anal. Biochem. 32, 420–424. 10.1016/S0003-2697(69)80009-6 5361396

[B43] Wang L. Li X. R. Lian H. Ni D. A. He Y. K. Chen X. Y.. (2010). Evidence That high activity of vacuolar invertase is required for cotton fiber and Arabidopsis root elongation through osmotic dependent and independent pathways, respectively. Plant Physiol. 154, 744–756. 10.1104/pp.110.162487 20699399 PMC2948991

[B44] Wind J. Smeekens S. Hanson J. (2010). Sucrose: metabolite and signaling molecule. Phytochemistry 71, 1610–1614. 10.1016/j.phytochem.2010.07.007 20696445

