# Supplementary material for: Effect of Waterlogging on Carbohydrate Metabolism and the Quality of Fiber in Cotton (Gossypium hirsutum L.)
Source: Front Plant Sci. 2016 Jun 22;7:877. doi: 10.3389/fpls.2016.00877 (PMC4916335; doi:10.3389/fpls.2016.00877)
Supplement: Figure S1 — The complete non-cropped image of the gels presented in Figure 5. [file Image1.PDF]

18S

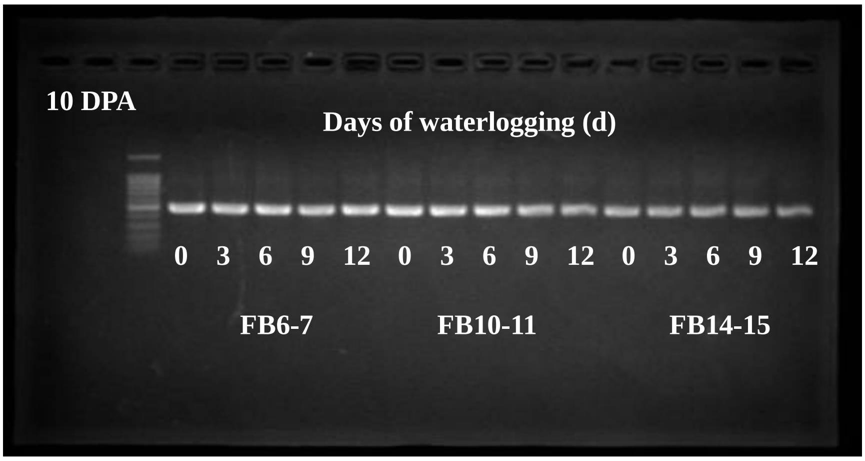
10 DPA
Days of waterlogging (d)
0 3 6 9 12 0 3 6 9 12 0 3 6 9 12
FB6-7
FB10-11
FB14-15

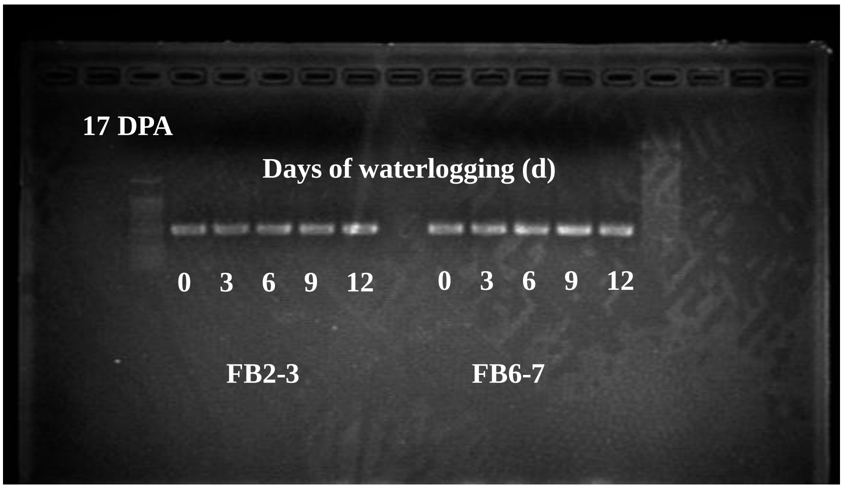
17 DPA
Days of waterlogging (d)
0 3 6 9 12 0 3 6 9 12
FB2-3
FB6-7

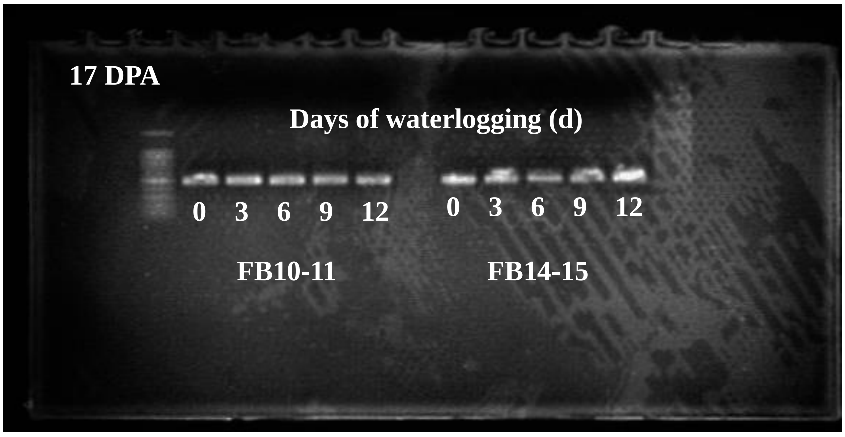
17 DPA
Days of waterlogging (d)
0 3 6 9 12 0 3 6 9 12
FB10-11
FB14-15

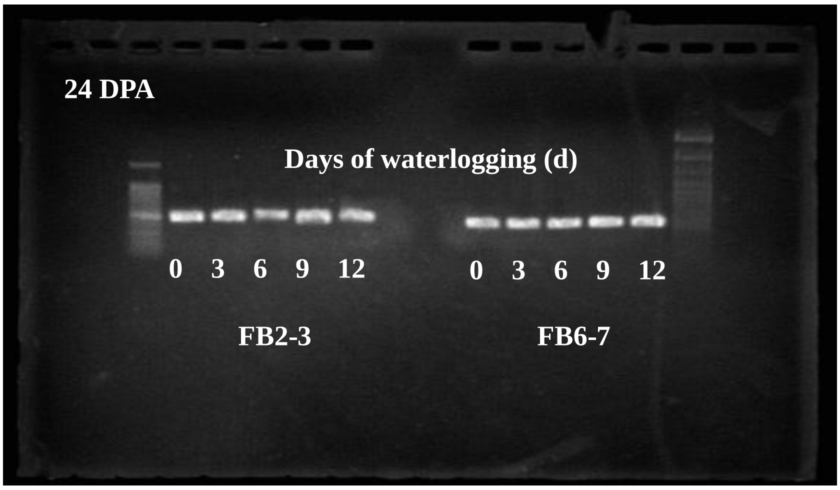
24 DPA
Days of waterlogging (d)
0 3 6 9 12
0 3 6 9 12
FB2-3
FB6-7

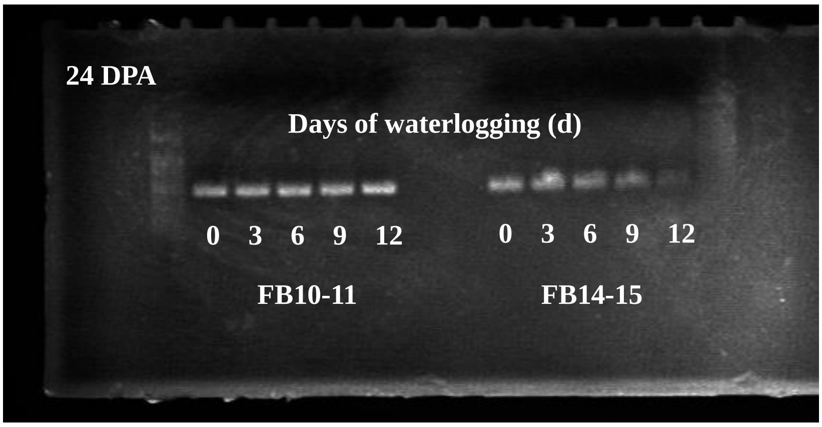
24 DPA
Days of waterlogging (d)
0 3 6 9 12
0 3 6 9 12
FB10-11
FB14-15

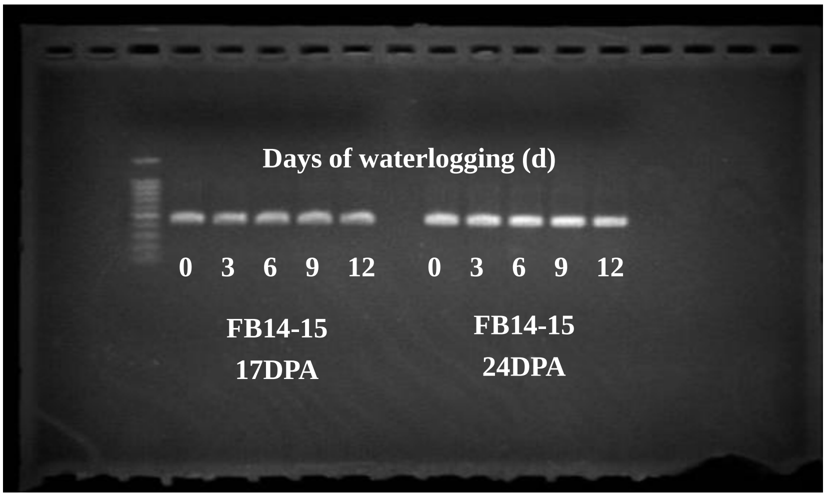
Days of waterlogging (d)
0 3 6 9 12
0 3 6 9 12
FB14-15
17DPA
FB14-15
24DPA

beta-1,3-glucanase

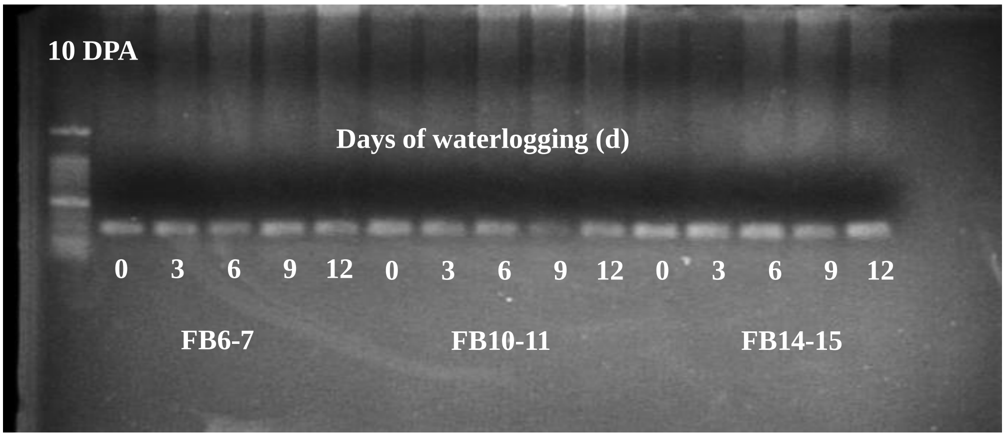

As *expressions of beta-1,3-glucanase in fibers at 17DPA and 24DPA were at very low level. So they are not presented here.*

beta-1,4-glucanase

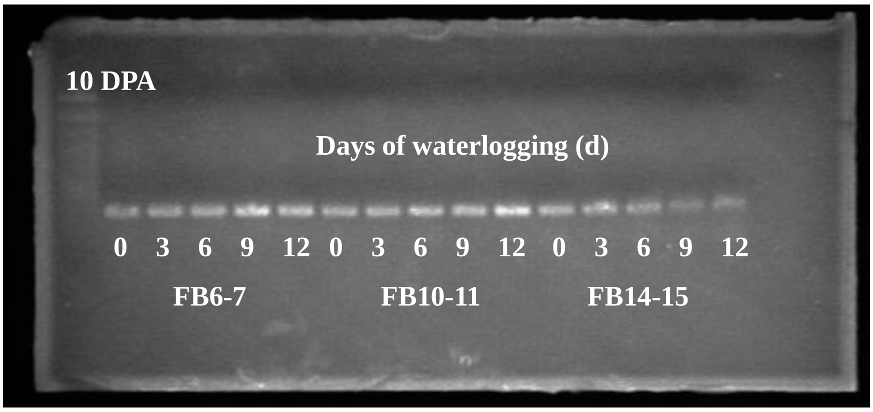

As *expressions of beta-1,4-glucanase in fibers at 17DPA and 24DPA were at very low level. So they are not presented here.*

Expansin

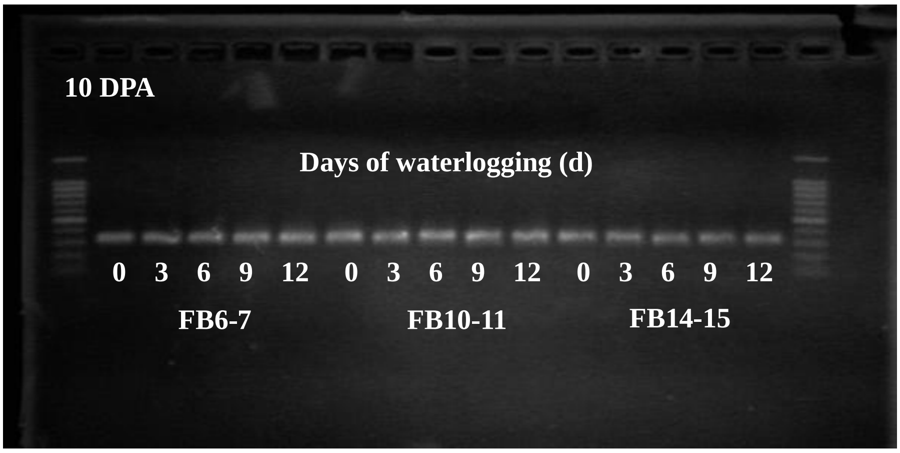
10 DPA
Days of waterlogging (d)
0 3 6 9 12 0 3 6 9 12 0 3 6 9 12
FB6-7
FB10-11
FB14-15

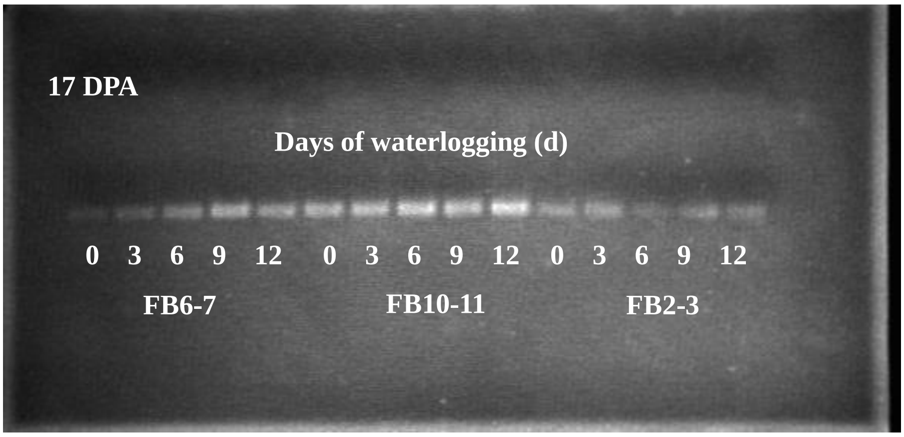
17 DPA
Days of waterlogging (d)
0 3 6 9 12 0 3 6 9 12 0 3 6 9 12
FB6-7
FB10-11
FB2-3

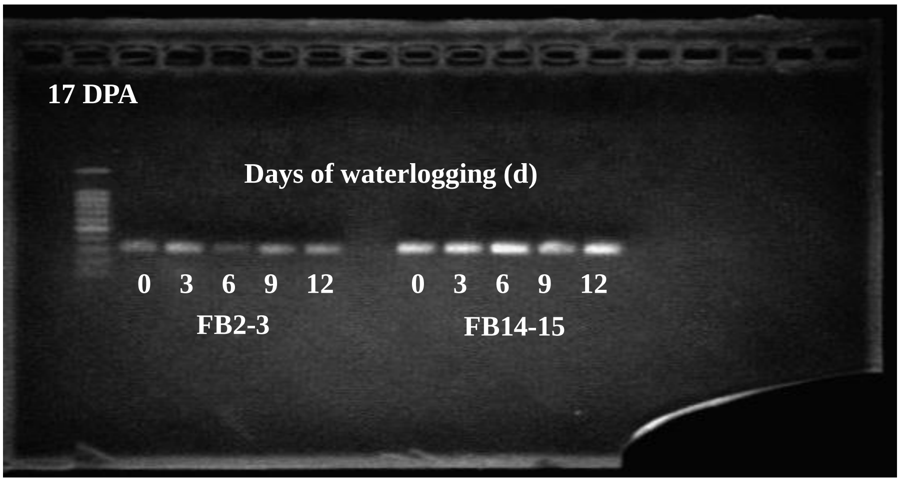
17 DPA
Days of waterlogging (d)
0 3 6 9 12 0 3 6 9 12
FB2-3
FB14-15

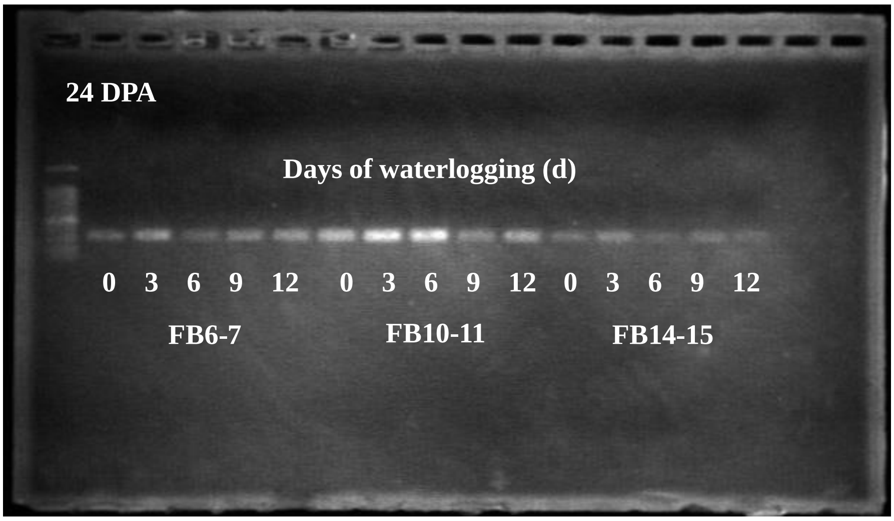
24 DPA
Days of waterlogging (d)
0 3 6 9 12 0 3 6 9 12 0 3 6 9 12
FB6-7
FB10-11
FB14-15

Invertase

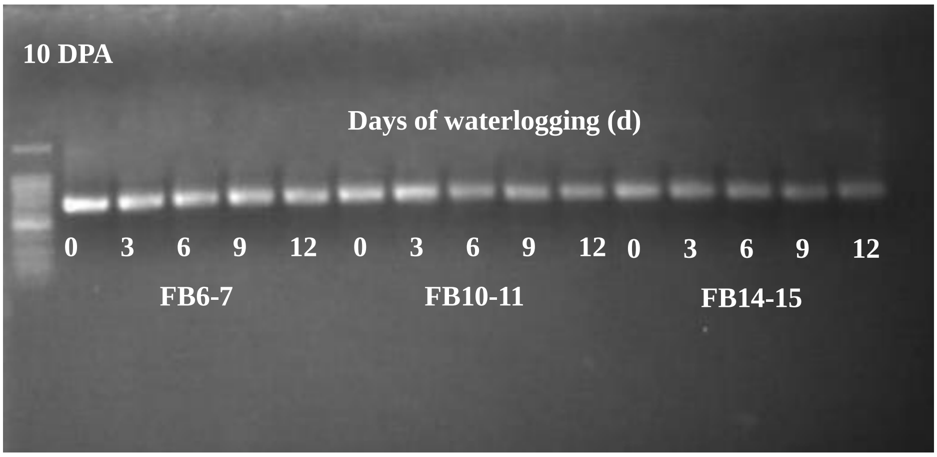
10 DPA
Days of waterlogging (d)
0 3 6 9 12 0 3 6 9 12 0 3 6 9 12
FB6-7
FB10-11
FB14-15

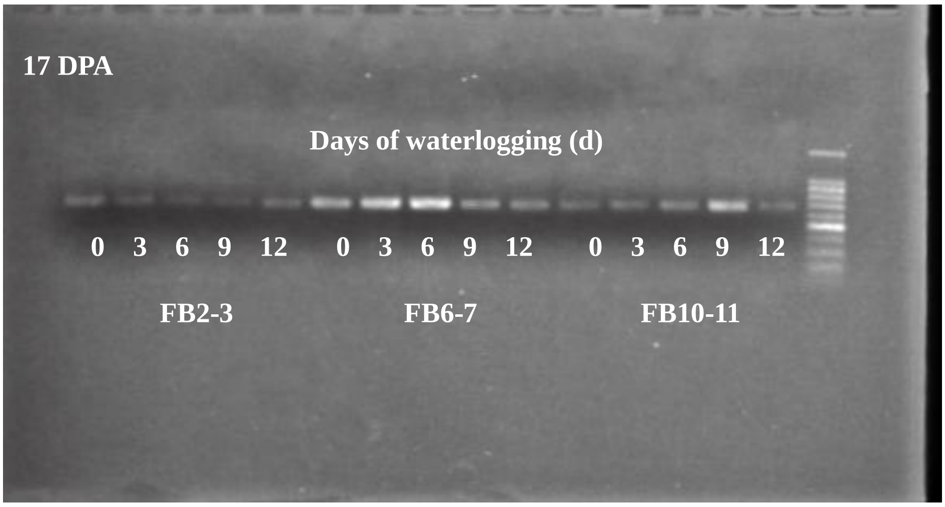
17 DPA
Days of waterlogging (d)
0 3 6 9 12 0 3 6 9 12 0 3 6 9 12
FB2-3
FB6-7
FB10-11

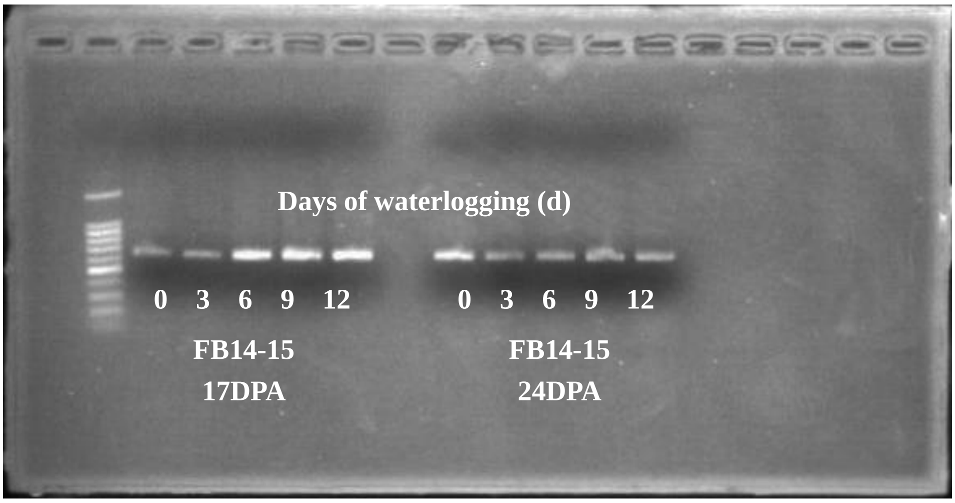
Days of waterlogging (d)
0 3 6 9 12 0 3 6 9 12
FB14-15
17DPA
FB14-15
24DPA

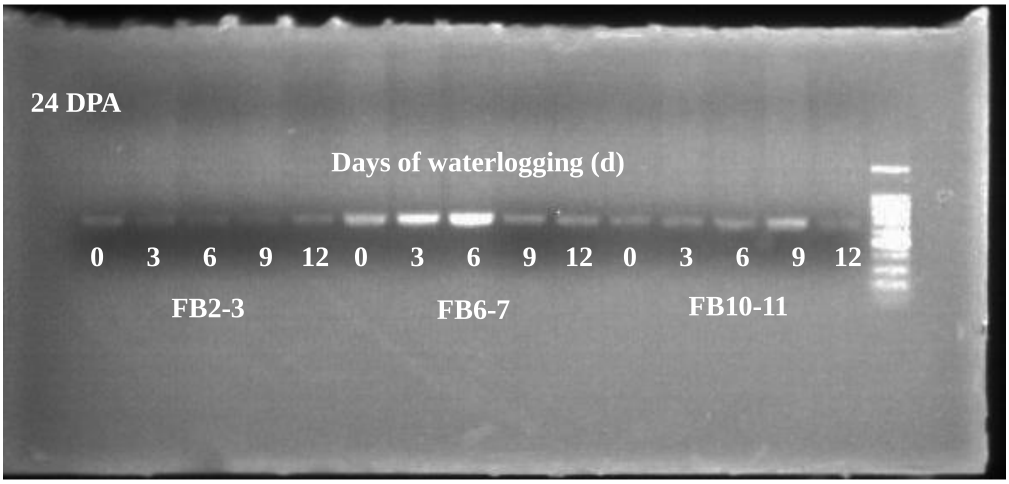
24 DPA
Days of waterlogging (d)
0 3 6 9 12 0 3 6 9 12 0 3 6 9 12
FB2-3
FB6-7
FB10-11

XET

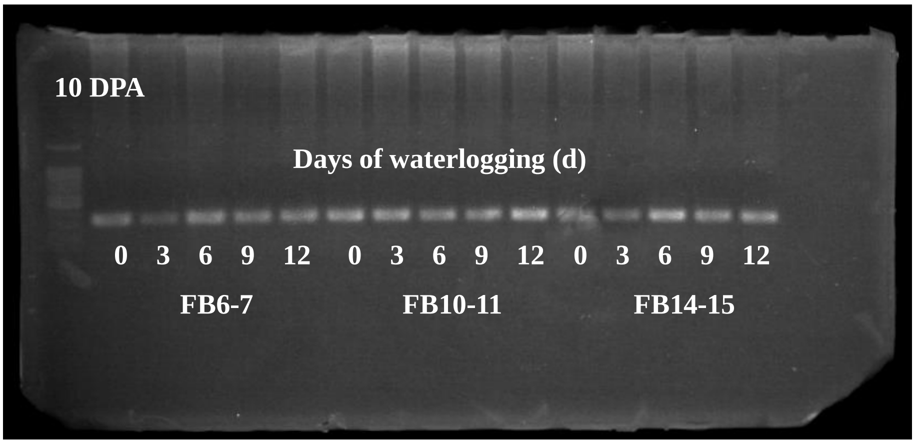
10 DPA
Days of waterlogging (d)
0 3 6 9 12 0 3 6 9 12 0 3 6 9 12
FB6-7
FB10-11
FB14-15

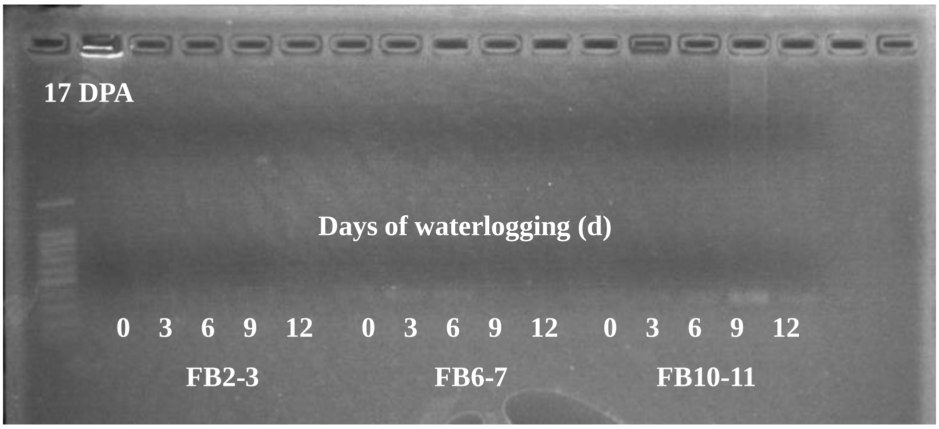
17 DPA
Days of waterlogging (d)
0 3 6 9 12 0 3 6 9 12 0 3 6 9 12
FB2-3
FB6-7
FB10-11

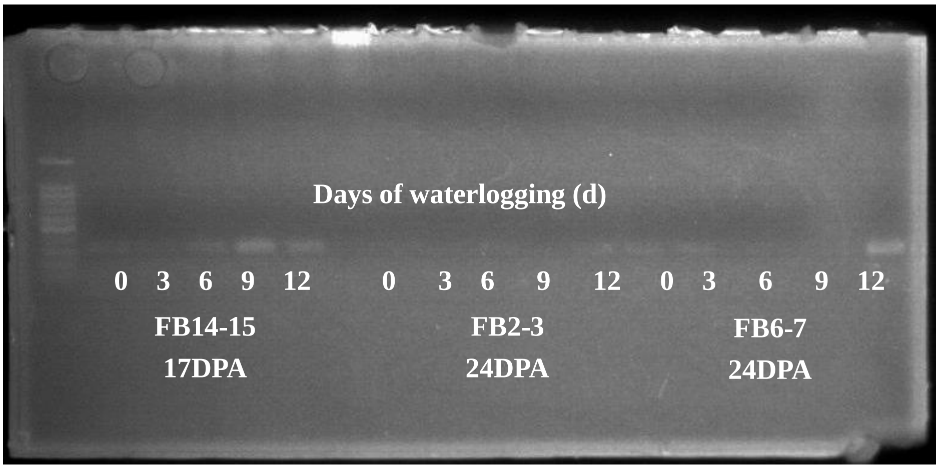
Days of waterlogging (d)
0 3 6 9 12 0 3 6 9 12 0 3 6 9 12
FB14-15
17DPA
FB2-3
24DPA
FB6-7
24DPA

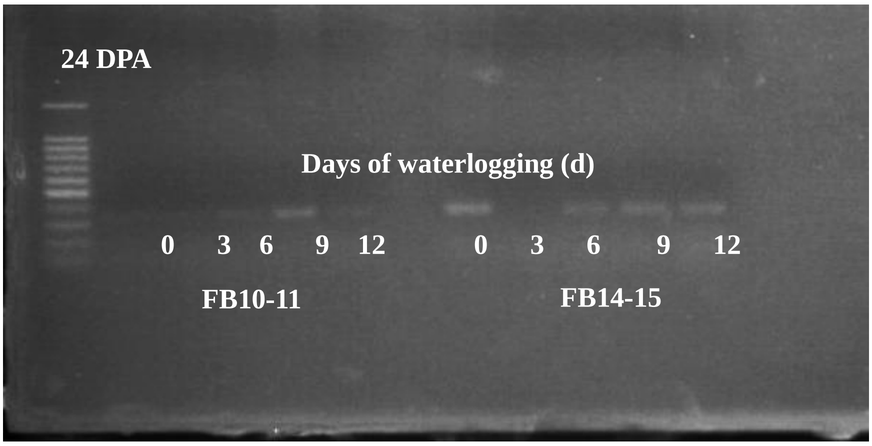
24 DPA
Days of waterlogging (d)
0 3 6 9 12
0 3 6 9 12
FB10-11
FB14-15
